# Promoting data harmonization to evaluate vaccine hesitancy in LMICs: approach and applications

**DOI:** 10.1186/s12874-023-02088-z

**Published:** 2023-11-24

**Authors:** Ryan T. Rego, Yuri Zhukov, Kyrani A. Reneau, Amy Pienta, Kristina L. Rice, Patrick Brady, Geoffrey H. Siwo, Peninah Wanjiku Wachira, Amina Abubakar, Ken Kollman, Akbar K. Waljee

**Affiliations:** 1 Center for Global Health Equity, Michigan Medicine, University of Michigan, Ann Arbor, MI USA; 2 Department of Epidemiology, School of Public Health, University of Michigan, Ann Arbor, MI USA; 3 Center for Political Studies, Institute for Social Research, University of Michigan, Ann Arbor, MI USA; 4 Edmund A Walsh School of Foreign Service, Georgetown University, Washington, DC USA; 5 Inter-university Consortium for Political and Social Research, Institute for Social Research, University of Michigan, Ann Arbor, MI USA; 6 Department of Learning Health Sciences, Michigan Medicine, University of Michigan, Ann Arbor, MI USA; 7 Institute for Human Development, Aga Khan University, Nairobi, Kenya; 8 Department of Political Science, College of Letters, Sciences, and Arts, University of Michigan, Ann Arbor, MI USA

**Keywords:** Data science, Vaccination, COVID-19, Political, Hesitancy, LMIC, Development, Elections

## Abstract

**Background:**

Factors influencing the health of populations are subjects of interdisciplinary study. However, datasets relevant to public health often lack interdisciplinary breath. It is difficult to combine data on health outcomes with datasets on potentially important contextual factors, like political violence or development, due to incompatible levels of geographic support; differing data formats and structures; differences in sampling procedures and wording; and the stability of temporal trends. We present a computational package to combine spatially misaligned datasets, and provide an illustrative analysis of multi-dimensional factors in health outcomes.

**Methods:**

We rely on a new software toolkit, Sub-National Geospatial Data Archive (SUNGEO), to combine data across disciplinary domains and demonstrate a use case on vaccine hesitancy in Low and Middle-Income Countries (LMICs). We use data from the World Bank’s High Frequency Phone Surveys (HFPS) from Kenya, Indonesia, and Malawi. We curate and combine these surveys with data on political violence, elections, economic development, and other contextual factors, using SUNGEO. We then develop a stochastic model to analyze the integrated data and evaluate 1) the stability of vaccination preferences in all three countries over time, and 2) the association between local contextual factors and vaccination preferences.

**Results:**

In all three countries, vaccine-acceptance is more persistent than vaccine-hesitancy from round to round: the long-run probability of staying vaccine-acceptant (hesitant) was 0.96 (0.65) in Indonesia, 0.89 (0.21) in Kenya, and 0.76 (0.40) in Malawi. However, vaccine acceptance was significantly less durable in areas exposed to political violence, with percentage point differences (ppd) in vaccine acceptance of -10 (Indonesia), -5 (Kenya), and -64 (Malawi). In Indonesia and Kenya, although not Malawi, vaccine acceptance was also significantly less durable in locations without competitive elections (-19 and -6 ppd, respectively) and in locations with more limited transportation infrastructure (-11 and -8 ppd).

**Conclusion:**

With SUNGEO, researchers can combine spatially misaligned and incompatible datasets. As an illustrative example, we find that vaccination hesitancy is correlated with political violence, electoral uncompetitiveness and limited access to public goods, consistent with past results that vaccination hesitancy is associated with government distrust.

**Supplementary Information:**

The online version contains supplementary material available at 10.1186/s12874-023-02088-z.

## Introduction

Analyses of health survey data often require linking surveys with information from other datasets on geographic contexts. There are many local social, economic and political factors relevant to health outcomes, but contextual data can be difficult to integrate with survey data, precluding interdisciplinary investigations. For example, a recent urgent problem has been to understand COVID-19 vaccine hesitancy in lower and middle income countries (LMICs). Vaccine hesitancy surveys often include questions about respondents' intent to vaccinate, exposure and beliefs surrounding COVID, and basic demographics. Some also include questions on household-level socioeconomic conditions and individuals' trust in the government. These surveys typically do not contain contextual and environmental information that may influence attitudes, like violence, elections or infrastructure. Data on these topics exist, but usually are from different sources, in different formats, and at different spatio-temporal levels of analysis, creating technical barriers to incorporating these data into analyses.

The Sub-National Geospatial Data Archive (SUNGEO) offers a new set of tools to address challenges of combining data with misaligned spatial units and boundaries, different geographic supports, data formats, and measurement strategies. SUNGEO’s goal is to reduce barriers to data integration, allowing researchers to probe the generalizability of empirical results to other geographic and historical contexts, and distinguish case-specific idiosyncrasies and short-term variation from broader trends and patterns. These open-source tools enable the transformation of data onto a common spatio-temporal scale, accounting for spatial misalignment, disharmonization, and differences in measurement. We create bespoke datasets and analyze data on vaccine hesitancy in three LMICs to illustrate how our approach can empower researchers to ask questions not answerable with a single data set, and facilitate assessments of generalizability and robustness.

This demonstration focuses on three contextual factors potentially relevant to vaccine hesitancy: political violence, electoral competitiveness, and economic development. We test three hypotheses: 1) there is more vaccination willingness where political violence is low; 2) there is more vaccine willingness where elections are highly competitive; and 3) there is more vaccine willingness where the local level of economic development is high. These hypotheses emerge from past literature, showing that exposure to violence tends to decrease trust in government institutions, that incumbents in more competitive seats have an incentive to implement effective public policies, and that mass vaccination campaigns are more difficult to implement in underdeveloped areas [1–4]. We test these hypotheses in Indonesia, Malawi, and Kenya, by integrating spatially misaligned datasets using the SUNGEO software package and data repository. This paper illustrates the use case for SUNGEO, and serves as a starting point for additional research on improving uptake of COVID-19 and other vaccines.

## Methods

Testing vaccine hesitancy hypotheses using multiple datasets presents challenges. Datasets associated with household-level and contextual factors have different geographic support, defined as the area, shape, size, and orientation of spatial measurement. Data on vaccine acceptance tend to come from household-level surveys; data on political violence are typically point-level event coordinates; data on elections tend to be measured by electoral constituencies (e.g. legislative districts); key development indicators, like road infrastructure, may be available as polyline features. These data come in different formats and structures (delimited text, vectors of location attributes, raster images); areal units are not nested and have misaligned borders; some of the data (e.g. surveys) may not be georeferenced at all. Different data integration choices may yield different results, raising concerns over generalizability [5]. Differences in sampling, question wording and sequence, primary sources, operational definitions, digital image processing algorithms, and other factors ensure that no two datasets are perfect substitutes for one another, making it difficult to distinguish case-specific idiosyncracies from general patterns, and to ask, "what does country A tell us about country B?" Finally, survey data pose a separate challenge of distinguishing "snapshots" of public attitudes from stable long-term trends. We illustrate how to mitigate some of these common challenges. The SUNGEO system accounts for these issues.

### SUNGEO

**Fig. 1:**
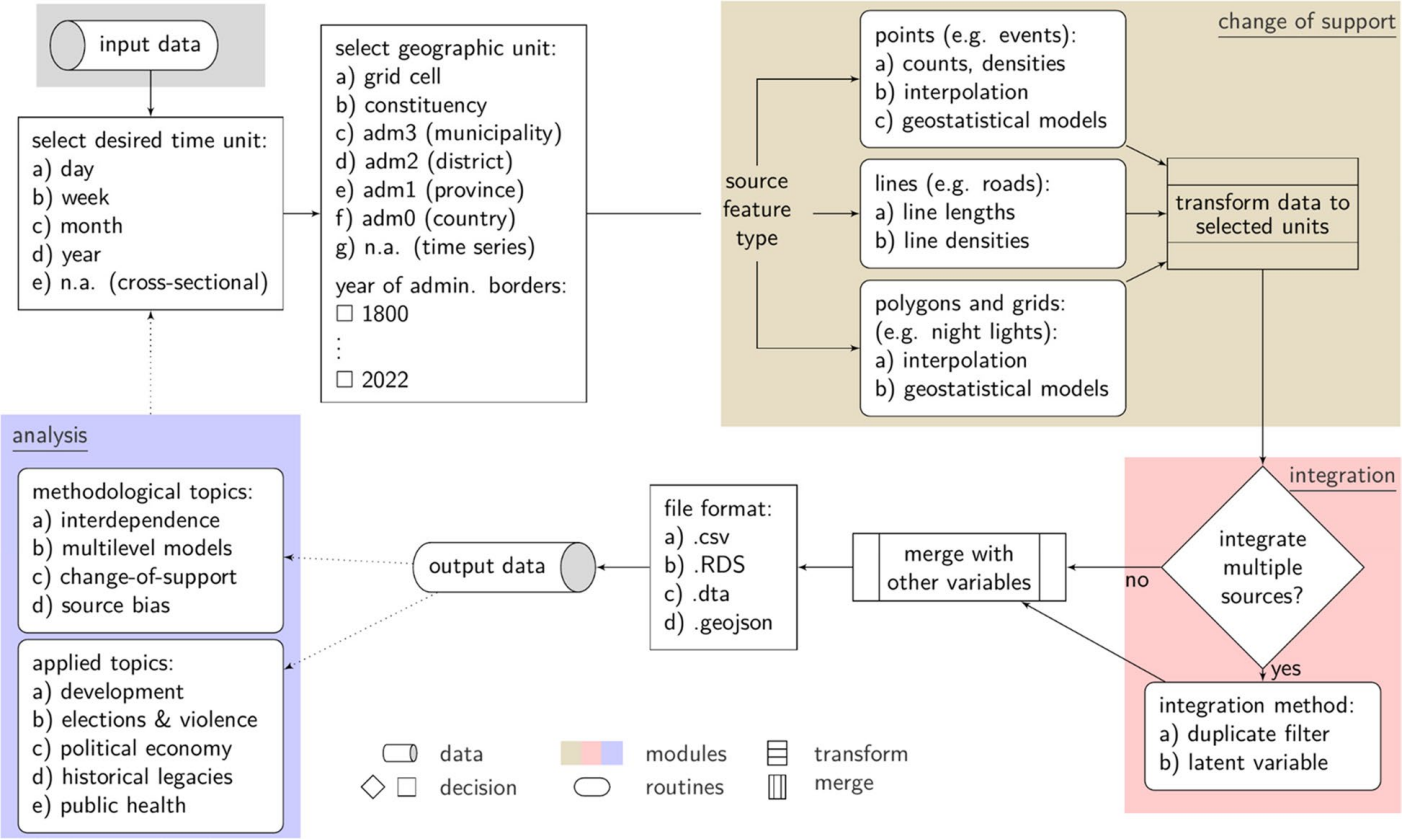
Overview of the SUNGEO system

SUNGEO allows users to combine data across otherwise incompatible geographic units into a common format, and facilitates the analysis and visualization of processed geospatial data (Fig. 1). It includes a user-friendly web interface and API, where researchers can select among many existing variables, choose levels and methods of spatiotemporal (dis)aggregation, interpolation and integration, and decide on the boundaries of their subnational datasets. Its large collection of pre-processed data enables users to replicate their research designs across different scales, data sources, countries, and integration procedures. SUNGEO also includes an open-source software package in the R statistical programming language to process user-supplied data, merge it with pre-loaded geo-referenced data, and produce a more customizable output based on user needs and specifications. It includes an archiving tool, which allows users to contribute original data to the repository.

### Description of the datasets

This demonstration uses vaccination hesitancy data from the World Bank Group’s High Frequency Phone Surveys (HFPS). The HFPS was a longitudinal cohort (panel) study on the socio-economic impacts of COVID-19 conducted in 53 countries and contexts between 2020 and 2022, with a subset of surveys including questions on vaccination hesitancy. We analyzed surveys from Indonesia, Kenya, and Malawi, as they: 1) were larger surveys with rigorous sampling methods representative of the general population; 2) included granular geographic information; and 3) were from three distinct regions (East Africa, Southern Africa, and South-East Asia). The survey datasets include sampling weights, based on the inclusion probabilities of the cell phones and landlines through which respondents were reached, along with first-time and attrition non-response weighting adjustments, and calibration with auxiliary information on regional population size, respondent sex, age group, and educational attainment. More information on each dataset can be found from the World Bank Group [6].

Contextual variables were provided from SUNGEO's preprocessed spatial data archive. Sub-national data on political violence are available for 195 countries through SUNGEO's partnership with the xSub data repository, which hosts leading event databases, including the Armed Conflict Location and Event Data Project (ACLED), the National Violence Monitoring System (NVMS), the Social Conflict Analysis Database (SCAD), and the Uppsala Conflict Data Program's Georeferenced Event Dataset (UCDP-GED). We chose among these by re-estimating our empirical models with each dataset on violence, and selecting the data source that yielded the strongest model fit (NVMS for Indonesia, UCDP-GED for Kenya, SCAD for Malawi; see Additional file 2: Appendix B3). Data on legislative elections in 168 countries are available through the Constituency-Level Elections Archive (CLEA). As a proxy measure for economic development, we used local road density, which can be calculated using the Global Roads Open Access Data Set (gRoads). More information on these datasets can be found from their respective sources [7–11]. We also used SUNGEO to extract data on other geographic variables that may affect attitudes toward, or the availability of, vaccines. These include ethno-linguistic fractionalization, average night light intensity, and terrain (see Additional file 2: Appendix B2 for details and estimation results) [12–14].

### Data curation process

- *a. Vaccine surveys*
  HFPS data are available through the Inter-university Consortium for Political and Social Research (ICPSR). ICPSR secured the World Bank’s permission to access HFPS data, then carried out a disclosure risk review to prevent direct or inferential re-identification of individuals or organizations. The curation process included generating question text employing the social science variables database to compare across studies,reviewing data to ensure all translations were correct and to create the variable and values list, conducting quality control, and hosting of the data on the ICPSR website in a fully searchable format. Further detail can be found in Additional file 1: Appendix A. In Additional file 2: Appendix B1, we examine sample attrition patterns across rounds, and find that respondents who dropped out of these samples were statistically similar on observables to those who remain.
- *b) Contextual data*
  Disaggregated data on violence, elections and economic development are available through SUNGEO. In aggregate form, the violence data are event counts, representing the number of incidents of political violence observed in each spatial unit over the two decades prior to the first survey. The election data are weighted averages of local "Top-1" competitiveness from the most recent legislative election, measured as one minus the winning vote margin, where values of 1 indicate that the most recent parliamentary election was very close, and 0 indicates that it was not competitive because the winner received almost all of the votes. We also considered alternative measures of electoral competitiveness, but the "Top-1" measure yielded a generally stronger model fit (Additional file 2: Appendix B3). The road density data are local sums of primary and secondary road lengths in each administrative unit, divided by that unit's area in square kilometers.

For each country, we used SUNGEO to extract data on political violence, legislative election data, and road infrastructure data, along with other contextual datasets (Additional file 2: Appendix B1). For Indonesia and Malawi, our spatial units were level-2 administrative divisions. For Kenya, we used level-1 administrative divisions.

To link data to household-level vaccine surveys, we used SUNGEO's R package to geocode survey sampling units, assigning a pair of geographic coordinates to each unique location. This allowed us to match each surveyed household to its corresponding level-2 (or level-1, in Kenya) spatial unit, and merge the datasets geographically (see Additional file 2: Appendix B1).

### Estimation strategy

We examined why some households express stable, pro-vaccine preferences, while others remain vaccine hesitant, or change their minds. Vaccine hesitancy varies spatially (across households) and temporally, with households changing their position. In the Indonesian survey, 73% of households gave the same answer to the vaccine intent question in two consecutive rounds (e.g. "yes" in rounds 4 and 5, or "no" in rounds 4 and 5). In Kenya, 68% gave the same answer across two rounds. In Malawi, 63% gave the same answer. Because the same households may give different responses on different occasions, we needed an empirical strategy that explicitly accounts for this shifting dynamic.

We modeled the survey responses as a stochastic process (Markov Chain) with two states. When asked the question, “if the vaccination was available for you at no cost, would you take the vaccination,” a household may either:

1. Express an intent to receive the Covid-19 vaccine ("yes"), or
2. Not express such an intent ("no").

From one round to another, a household will have some probability of staying with their previous response, and some probability of transitioning to another response. We model these transition probabilities as conditional on a series of household-level and contextual covariates:

1 $$\text{Pr}(\mathrm{y}_{\mathrm{i},\mathrm{t}}\;=\;1)\;=\;\text{logit}^{-1}\;\left[\mathrm{x}_{\mathrm{i}}{{\theta}}_{0}\;+\;{\mathrm{y}}_{\mathrm{i},\mathrm{t}-1}\;\cdot\;{\mathrm{x}}_{\mathrm{i}}\upgamma\;+\;{\alpha}_{{\mathrm{k}}_{(\mathrm{i})}}+\;{\tau}_{\mathrm{t}}\;+\;{\varepsilon}_{\mathrm{i},\mathrm{t}}\right]$$

where *y*_i,t_ is 1 if household *i* says "yes" in round *t*, and 0 if the household says "no", *y*_i,t-1_ is a first-order temporal lag, α_k(i)_ is a fixed effect for the administrative unit *k* in which *i* is located, τ_t_ is a fixed effect for each survey round, and ε_i,t_ are robust standard errors, clustered by administrative unit and survey round. The vector of covariates **x**_i_ includes household-level measures like respondent's age and gender, and an indicator for whether the household is located in an urban area, as well as contextual information on violence, electoral competitiveness, road density, night light intensity, ELF, and terrain.

θ_0_ are regression coefficients for households that said "no" to the vaccine at *t-1*, and θ_1_ = θ_0_ + ɣ are coefficients for households that said "yes" at *t-1*. We will use these coefficient estimates to generate predicted probabilities of vaccine intent, and to construct transition probability matrices.

We estimated the model in Eq. (1) separately on integrated survey datasets from Indonesia, Kenya and Malawi.

## Results

The full set of summary statistics, coefficient estimates and simulation results are in Appendix B2.

### Probabilities of vaccine intent transition in Indonesia

Table 1 shows a transition probability matrix for a median Indonesian household, based on the estimated parameters of the model in Eq. (1).^1^ Additional file 2: Appendix B2 reports analogous tables for the other two countries. Most households are likely to stay with their previous answer, particularly if that answer was "yes" (i.e. willing to take the vaccine). All else equal, 35% of households that said "no" in round *t-1* are predicted to say "yes" in round *t*. Meanwhile, just 4% of households that said "yes" in round *t-1* are predicted to switch to "no" in the next round.

**Table 1:** Transition probabilities for a median Indonesian household

	Answer in current round:	
Answer in previous round:	"No"	"Yes"
"No"	0.65	0.35
"Yes"	0.04	0.96

Each cell in the matrix conveys the probability of transitioning from one state at time *t-1* (in the rows) to another state at time *t* (in the columns). The diagonal cells represent probabilities of staying in either the "no" or "yes" state; off-diagonal cells represent probabilities of switching from "no" to "yes" (upper right) or "yes" to "no" (lower left)

Through an eigenvalue decomposition of this transition matrix, we can obtain the stationary distribution of vaccination intent for a median Indonesian household. Over time, 90% of households will commit to an answer of "yes", and 10% will commit to an answer of "no". We found similar stationary distributions in Kenya (0.88 to 0.12) and Malawi (0.72 to 0.28) (Additional file 2: Appendix B2).

The results reported so far apply to households located in a median administrative unit (i.e. median levels of violence, electoral competitiveness, road density, etc.). To test our three hypotheses, and assess how these long-run probabilities change under different local conditions, we re-estimate the stationary distribution under counterfactual scenarios.

#### Violence

**Fig. 2:**
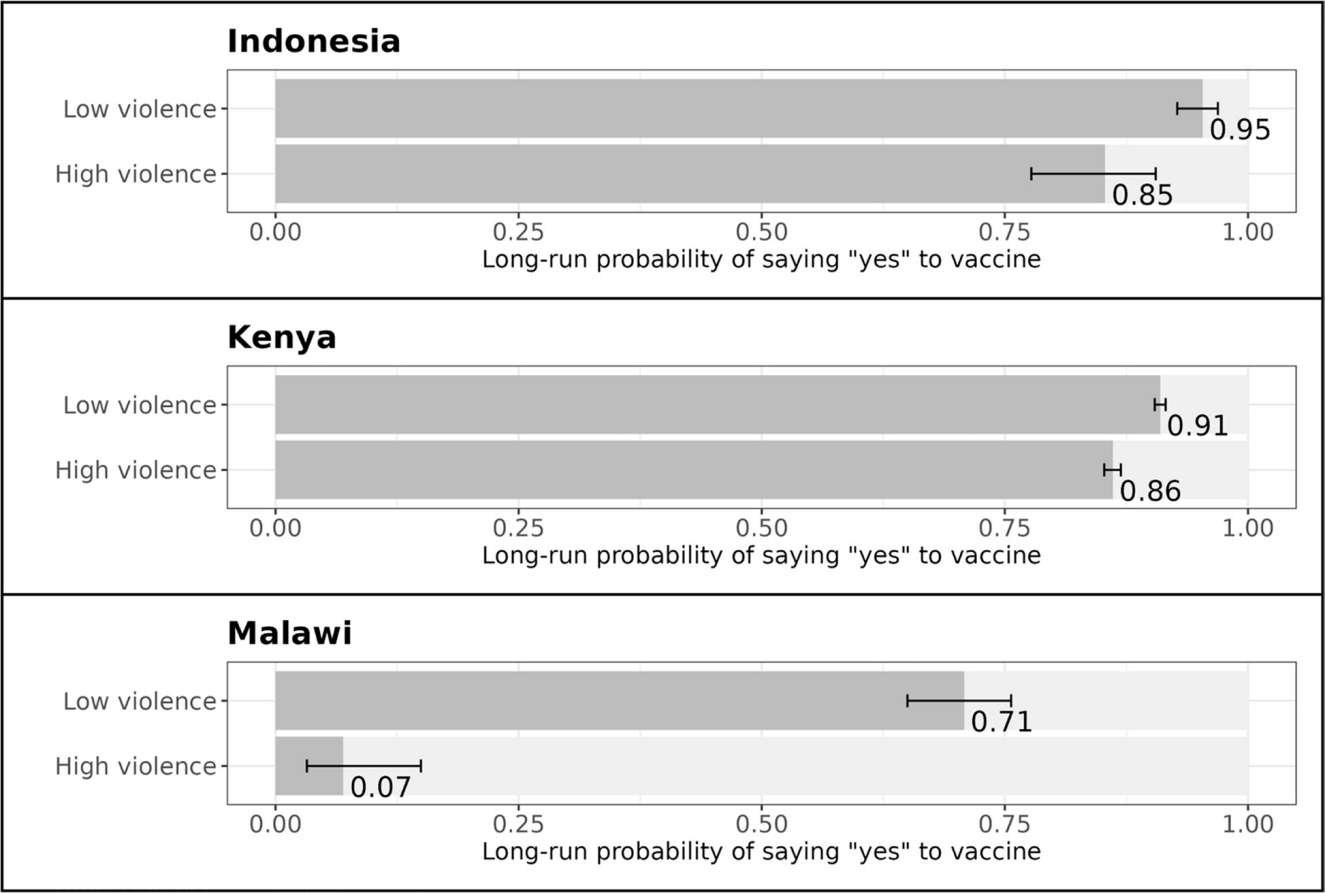
Exposure to violence and vaccine hesitancy. Dark gray bars represent proportions of respondents predicted to commit to an answer of "yes" on the vaccine intent survey question. Horizontal brackets represent bootstrapped 95% confidence intervals

For the first hypothesis, the predicted stationary distributions in Fig. 2 suggest that households with a greater potential exposure to violence are more vaccine hesitant. In all three countries, the long-run probability of saying "yes" to the vaccine intent question is significantly lower among households in high-violence locations, and higher in low-violence locations. Indonesian households in high-violence areas (99th percentile) have an 85% chance of saying "yes"; in low-violence areas (1st percentile), the number for "yes" rises to 95%. In Kenya, households in high-violence areas have a 86% chance of saying "yes", while households in low-violence areas have a 91% chance of saying "yes". In Malawi, the gap is even wider: 7% versus 71%. In each case, these differences are statistically significant at the 95% confidence interval.

#### Elections

**Fig. 3:**
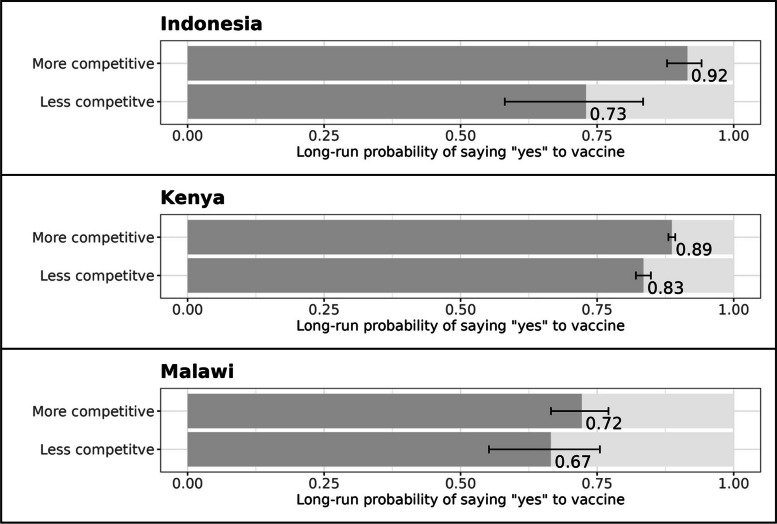
Electoral competitiveness and vaccine hesitancy

The stationary distributions in Fig. 3 are largely supportive of the second hypothesis. In Indonesia, households in less-competitive areas (1st percentile) have a 73% chance of saying "yes" to the vaccine in the long-run; in more competitive locations (99th percentile), the "yes" estimate rises to 92%. In Kenya, the "yes" numbers are 83% in less-competitive locations and 89% in more-competitive ones. In both countries, these are significant at the 95% confidence interval. In Malawi, the differences are in the same direction –- 67% "yes" in less-competitive locations, 72% in more-competitive ones –- but the large standard errors prevent us from drawing definitive conclusions.

### Economic development

**Fig. 4:**
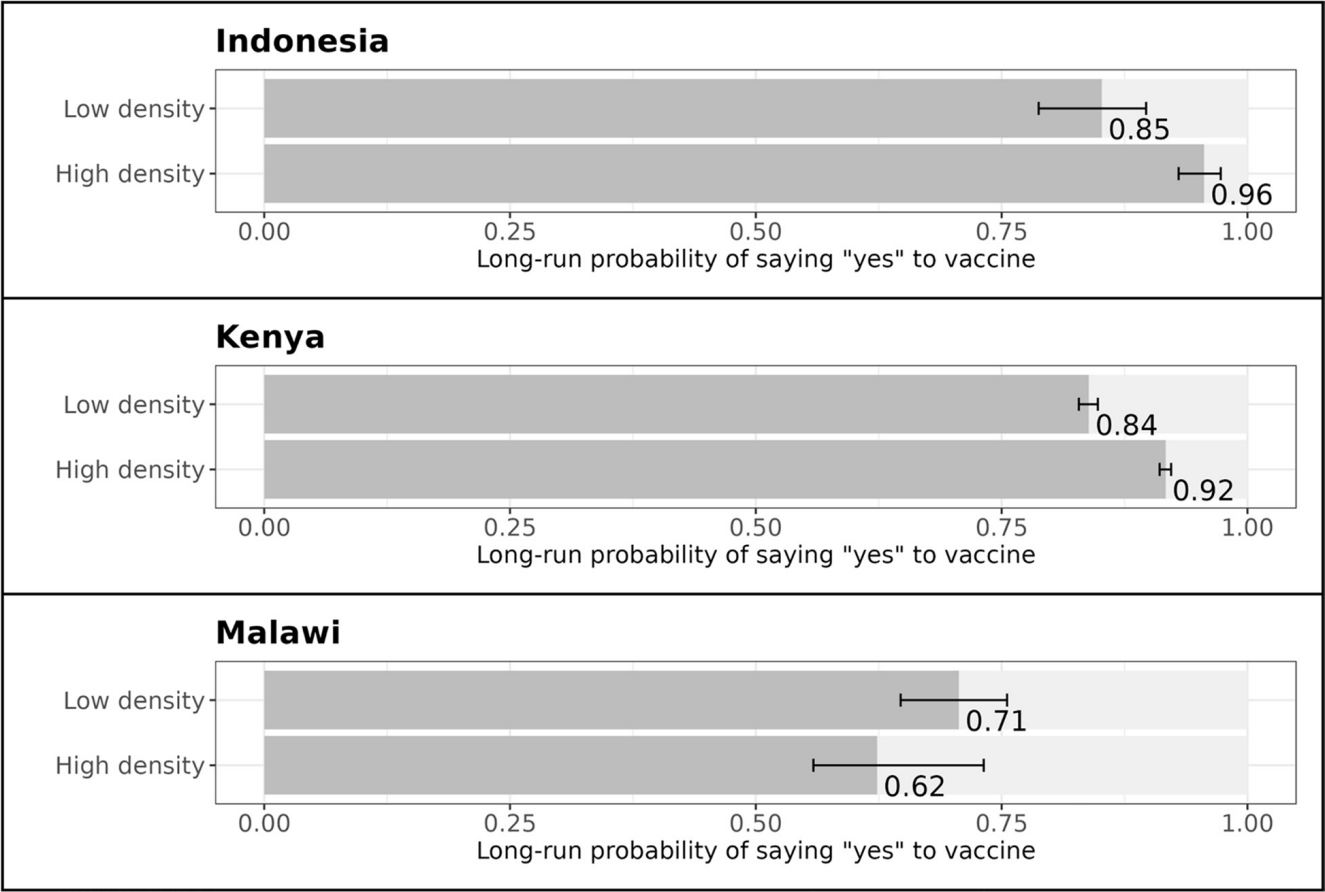
Economic development (road density) and vaccine hesitancy

The results in Fig. 4 are generally consistent with our final hypothesis. In the counterfactual stationary distributions for Indonesia, 96% of households in high road density areas (99th percentile) are expected to say "yes", compared to 85% in low road density areas (1st percentile). In Kenya, the estimates are 92% "yes" in high density areas, and 84% "yes" in low density areas. In Malawi the difference is statistically insignificant.

In Additional file 2: Appendix B2, we report similar counterfactual stationary distributions for all other covariates included in our models. In all three countries, the long-run probability of saying "yes" to the vaccine is lower for older respondents and for respondents who live in more ethnically fractionalized areas. Results for other covariates, like sex and urbanization, are more variable across countries.

## Discussion

While the goal of our analyses is to demonstrate the capabilities of SUNGEO, rather than establish causality, the results from our study bear notice. In all three countries, contextual factors relate to trajectories of vaccine hesitancy among households. Households were less likely to be vaccine acceptant, and likely to become even more vaccine hesitant over time, if their local administrative area had experienced high levels of political violence in recent years. In Kenya and Indonesia, households in areas where elections are tightly contested are more likely to be vaccine acceptant initially, and more likely to move away from vaccine hesitancy over time. Economic development also correlates with greater vaccine acceptance in Indonesia and Kenya, although we find no significant association in Malawi.

### The impact of violence, electoral competitiveness and development on vaccination hesitancy

This study is unique in that it enables potential insight into the association between local contextual factors of violence, electoral competitiveness, development and vaccination hesitancy. Our findings are consistent with other research in Kenya on the role of institutional trust, which found that increased government trust was associated with decreased vaccination hesitancy [15]. In Western Europe, Kennedy (2019) found an association between vaccine hesitancy and distrust of political elites and experts, complementary to our findings in Kenya. Past studies have also found associations between vaccination hesitancy and socioeconomic status, which we may expect to be higher, on average, among households who reside in more economically developed areas [16, 17]. Past research in political science has shown that exposure to violence can undermine individuals' confidence in the government, while competitive elections make incumbents more publicly accountable, and more developed infrastructure can improve the delivery of public services [3, 18, 19].

To the extent that violence, elections and development have downstream implications for government trust, and mistrust in government can heighten vaccine hesitancy, our findings carry several policy implications. While institutional trust is not easily manipulated by policymakers, knowing where this trust might be most lacking can help policymakers more efficiently allocate scarce resources. Looking beyond targeted public information campaigns, our findings suggest that general efforts to decrease violence, increase electoral competitiveness, and expand transportation infrastructure may have positive externalities for vaccine acceptance.

It is important to consider why associations between local context and vaccine hesitancy are weaker in Malawi than in Indonesia or Kenya. Our analysis utilized the same data sources across countries, the same geospatial transformation methods, the same operationalizations and scales of key variables, the same model specification, and –- with the exception of Kenya –- the same level of administrative units. While our within-country analyses were not perfect clones of each other, SUNGEO allowed us to hold these specific elements of research design constant, and exclude them as potential sources of the disparity. There were some key differences: 1)survey data on Malawi had a significantly smaller sample size of 596 unique households, compared to 1847 for Indonesia and 7616 for Kenya, this reduced statistical power; 2) the Malawi panel survey had fewer rounds of questions about vaccine hesitancy: 2, compared to 4 and 5 for Kenya and Indonesia, so there is less information in the Malawi data about the evolution of the stochastic process over time; 3) there may be important unobserved differences in each local context, for which our estimation strategy did not fully account.

### Limitations

There are limitations of this analysis and method, and we suggest several steps to address these. First, survey questions on vaccination hesitancy were hypothetical. In Indonesia, 68% of households who expressed intent to be vaccinated in one round did not report being vaccinated by the next round.^2^This number, however, may reflect difficulties in accessing the vaccine, not only lack of follow-through on the part of respondents. Second, social desirability bias may have caused respondents to misrepresent their true intent, especially in later rounds of the survey [20]. While phone surveys are less susceptible to this type of bias than face-to-face surveys, they cannot rule it out entirely [21]. This potential bias may push in either direction –- overstating the intent in some cases, and understating it in others –- and its impact on our inferences is not immediately clear. Third, because enumerators typically interview the head of household, our analysis rests on the assumption that heads of household make vaccination decisions on other family members' behalf. Notably, we find no evidence that male heads of household differ systematically from female heads of household in their responses to this survey question. We explore these further in an accompanying paper [22].

A key limiting factor in our analyses is the geographic precision of survey sampling units. As we only observe the name of the administrative unit in which households reside, we cannot utilize variation in SUNGEO contextual variables within these units, and cannot account for more disaggregated community and neighborhood-level effects. Our inferences are also limited by the geographic scope of HFPS surveys, which did not reach many administrative units, particularly those in rural, underdeveloped areas. While our regression analyses utilized survey weights to make the samples more representative of national populations, such reweighting cannot facilitate inferences in locations where no data exist. To correct the geographic "blind spots", future research should explore tools that use national surveys to estimate public opinion in small areas and subpopulations that are undersampled or underrepresented, including multilevel regression and poststratification (MRP) [23]. Although, it is important to note that these results are not generalizable beyond the countries analyzed, future research could use SUNGEO to conduct similar analyses on additional countries.

Finally, we designed our estimation strategy around a very particular empirical phenomenon: stability and change in household survey responses over time. While our stochastic model can account for some of these shifting dynamics, it is ill-suited for other types of empirical inquiries, like causal identification and causal mediation analysis –- both of which are natural priorities for future research. In Additional file 2: Appendix B3, we report a battery of supplementary analyses, to evaluate the sensitivity of our results to spatial autocorrelation, general forms of cross-sectional and temporal dependence, selection bias, alternative data sources and measures, and additional cross-level interactions between respondent attributes (e.g. age, sex) and contextual factors (e.g. violence).

## Conclusion

We have introduced SUNGEO as a platform for integrating data across incompatible formats and units into analysis-ready datasets. This approach can overcome critical barriers in the analysis of contextual effects on health decisions, including differences in measurement and data sources across countries. SUNGEO offers a means to relieve such bottlenecks, and to examine whether particular integration and transformation methods matter for downstream results.

We invite further research to explore the generalizability of our findings. This paper presents an illustration of what is possible with a data infrastructure like SUNGEO. There are many other health outcomes that can be studied using these techniques, including preventive healthcare, malnutrition, and disparities in service and access. The results here reveal patterns in data that we could not discover without the means to combine novel data in rigorous ways.

Future work related to vaccine hesitancy could explore mechanisms behind why households respond to their contexts in the ways we have observed, and could be enhanced by including additional countries and additional types of health decisions. The former would require additional theories as to why health behaviors vary across political contexts. The latter would broaden the scope of the analyses to generalize key patterns in health outcomes. We encourage research expanding the depth and breadth of these inquiries.

### Supplementary Information

**Additional file 1.**

**Additional file 2.** 

## Data Availability

Data are publicly available from the World Bank Group or ICPSR. For ease, we have hosted the data as well at doi.org/10.17605/OSF.IO/FY4GN.

## Footnotes

1 The values are predicted probabilities from model (1), setting all covariates to their median values.

2 We excluded households from estimation in survey rounds when they reported taking the vaccine, since the vaccine intention question was not asked of these respondents.

## References

[1] Grosjean P (2014). Conflict and social and political preferences: evidence from world war ii and civil conflict in 35 European Countries. Comp Econ Stud.

[2] Corbetta P. Morris P. Fiorina, Retrospective Voting in American National Elections, New Haven-London, Yale University Press, 1981, pp. 249. (s.p.). Italian Political Science Review / Rivista Italiana di Scienza Politica 1982;12:479–81.

[3] Gordon, Huber. The effect of electoral competitiveness on incumbent behavior. Quart J Polit Sci. https://papers.ssrn.com/sol3/papers.cfm?abstract_id=1335455.

[4] Min BK-H. Democracy and Light: Public Service Provision in the Developing World. University of California, Los Angeles, 2010.

[5] Zhukov YM, Byers JS, Davidson MA, Kollman K. Integrating Data Across Misaligned Spatial Units. Polit Anal 2023; : 1–17.

[6] World Bank Microdata. https://microdata.worldbank.org/index.php/home (Accessed 19 Oct 2022).

[7] Zhukov YM, Davenport C, Kostyuk N (2019). Introducing xSub: A new portal for cross-national data on subnational violence. J Peace Res.

[8] Raleigh C, Linke A, Hegre H, Karlsen J (2010). Introducing ACLED: An armed conflict location and event dataset: special data feature. J Peace Res.

[9] Sundberg R, Melander E (2013). Introducing the UCDP Georeferenced Event Dataset. J Peace Res.

[10] CLEA. https://electiondataarchive.org/ (Aaccessed 28 Sept 2022).

[11] Global roads. https://sedac.ciesin.columbia.edu/data/set/groads-global-roads-open-access-v1 (Accessed 29 Sept 2022).

[12] NOAA national centers for Environmental Information (NCEI). 2012; published online Feb 10. https://www.ngdc.noaa.gov/ (Accessed 29 Sept 2022).

[13] Wucherpfennig J, Weidmann NB, Girardin L, Cederman L-E, Wimmer A (2011). Politically Relevant Ethnic Groups across Space and Time: Introducing the GeoEPR Dataset. Confl Manag Peace Sci.

[14] Hsu F-C, Baugh KE, Ghosh T, Zhizhin M, Elvidge CD (2015). DMSP-OLS Radiance Calibrated Nighttime Lights Time Series with Intercalibration. Remote Sensing.

[15] Rego RT, Ngugi AK, Delius AJS (2022). COVID-19 vaccine hesitancy among non-refugees and refugees in Kenya. PLOS Global Public Health.

[16] Larson HJ, Jarrett C, Eckersberger E, Smith DMD, Paterson P (2014). Understanding vaccine hesitancy around vaccines and vaccination from a global perspective: a systematic review of published literature, 2007–2012. Vaccine.

[17] Hudson A, Montelpare WJ. Predictors of Vaccine Hesitancy: Implications for COVID-19 Public Health Messaging. Int J Environ Res Public Health 2021;18. 10.3390/ijerph18158054. 10.3390/ijerph18158054 PMC8345367 34360345

[18] Walden J, Zhukov YM. Historical legacies of political violence. In: Oxford Research Encyclopedia of Politics. 2020.

[19] Burnett CM, Kogan V (2017). The politics of potholes: service quality and retrospective voting in local elections. J Polit.

[20] Rego R, Watson S, Gill P, Lilford R (2022). The impact of diarrhoea measurement methods for under 5s in low- and middle-income countries on estimated diarrhoea rates at the population level: A systematic review and meta-analysis of methodological and primary empirical studies. Trop Med Int Health.

[21] Rego R, Watson S, Ishengoma P, Langat P, Otieno HP, Lilford R (2020). Effectiveness of SMS messaging for diarrhoea measurement: a factorial cross-over randomised controlled trial. BMC Med Res Methodol.

[22] Rego RT, Reneau K, Zhukov Y, *et al.* Evaluating self-reported vaccination hesitancy in mobile phone surveys performed in LMICs: Learned lessons from data in four low and middle income countries. Research Square. 2022; published online Dec 17. 10.21203/rs.3.rs-2326701/v1.

[23] Gelman A, Little TC. Poststratification into many categories using hierarchical logistic regression. 1997. http://citeseerx.ist.psu.edu/viewdoc/summary?doi=10.1.1.44.5270.

