# Supplementary material for: Promoting data harmonization to evaluate vaccine hesitancy in LMICs: approach and applications
Source: BMC Med Res Methodol. 2023 Nov 24;23:278. doi: 10.1186/s12874-023-02088-z (PMC10668461; doi:10.1186/s12874-023-02088-z)
Supplement: Supplementary file 1 — Additional file 1. [file 12874_2023_2088_MOESM1_ESM.pdf]

Appendix A: Data Curation

In order to obtain the data from the World Bank Group's collection of High-Frequency Phone Surveys, ICPSR applied for access to the datasets by describing the intended use for the data and agreeing to terms and conditions. Next, an ICPSR staff person manually deposited the data, accompanying documentation and available metadata through ICPSR's secure deposit form. All data submitted to ICPSR undergo disclosure risk review to prevent direct or inferential re-identification of individuals or organizations, and to determine appropriate access level for the study (public, restricted, or a combination of both).

To maximize potential for secondary use, ICPSR curated these data at a Level 3 curation—the highest and most intensive level. Notably, Level 3 includes the creation of question text. This involves utilizing the literal questions asked of participants to generate a text document. This level also employs the Social Science Variables Database (SSVD)[22]. The SSVD permits secondary-users to search and compare variables across studies. These two speciality items help to enhance data exploration and usability facilitated by ICPSR's data curation process.

The first phase of the curation process is the "Review" phase. The curator begins reviewing the deposited materials and runs commands and scripts to check for foreign language characters, to provide basic variable and case counts, and to create a value and variable list. In addition to reviewing the data for disclosure risk, during this phase the curator will look for missing or incomplete information, and discrepancies between submitted data and documentation; this initial review culminates in a documented "Processing Plan." Based on the review of the data, a copy of the data is changed and enhanced. This is known as the "Processing" phase. Because curation is not a linear process, reviews of data changes are done often, including at this phase. Once the SPSS command script runs with no errors or unexpected warnings, we move into the "Production of Full Product Suite" phase. Here, Hermes—ICPSR's batch processing system— is run to produce the full product suite. This suite includes: A stat file for each of the four statistical packages (SAS, SPSS, Stata, R), a flat text file (ASCII) and a tab delimited file, setup files for SAS, SPSS, and Stata, a DDI file, and a PDF frequencies codebook. All documentation produced during this stage, for example, the ICPSR codebook, are released as PDF files and follow strict standards for accessibility. Other documentation may include user guides, or questionnaires.

It is important to note that during all stages of the curation process, there are various rounds of quality control (QC) performed by various curators. Once a study has passed all required rounds of QC and is approved for turnover, the "Turnover Process" occurs. This means that the study is ready to be "turned over" and released to the ICPSR website. The final phase is called "Post-release Checks." Once turnover is complete, the curator performs "post-turnover checks" which involves verifying that the study appears on the website as intended. Checks are performed on the data, documentation, metadata, and any supplemental files.

The curation of the studies for this particular project resulted in a series page, or a dedicated webpage that groups related studies together. Usage metrics are available on each study

homepage to easily track the number of downloads, as well as the profile of those utilizing the data.

ICPSR follows best practices for the long-term preservation of data. Users can find data by using our search page or Google since ICPSR uses the schema.org standard to make structured metadata available. Additionally, ICPSR makes metadata records available via the Open Archives Initiative Protocol for Metadata Harvesting (OAI-PMH), which allows academic libraries and vendors to include them in online public access catalogs. Finally, each study is assigned a DOI (digital object identifier), which is a persistent link to the study that can be used in citations. The DOI ensures that the study can be easily linked over time. As part of the DOI registration process, ICPSR shares the study metadata with our DOI registrar, Datacite, who then ingests the information into their catalog, [23].

Following this procedure, ICPSR curated vaccine survey data from the World Bank Group for Indonesia, Kenya and Malawi. Each is a panel dataset with repeated observations per household, across multiple survey rounds. The Indonesian survey included 2846 households in 4 rounds over the period November 2020 to April 2022.[1] The primary sampling units were level-2 administrative divisions. The Kenyan survey included 5559 households in 4 rounds over the period May 2020 to July 2022. The primary sampling units were level-1 administrative divisions. The Malawi survey included 795 households in 2 rounds over the period May 2020 to May 2022. The primary sampling units were level-2 administrative divisions.

---

[1] This number includes households who answered the vaccine hesitancy survey question, and have non-missing values for all covariates.
