# Supplementary material for: Promoting data harmonization to evaluate vaccine hesitancy in LMICs: approach and applications
Source: BMC Med Res Methodol. 2023 Nov 24;23:278. doi: 10.1186/s12874-023-02088-z (PMC10668461; doi:10.1186/s12874-023-02088-z)
Supplement: Supplementary file 2 — Additional file 2. [file 12874_2023_2088_MOESM2_ESM.pdf]

Appendix B:

Promoting Data Harmonization to Evaluate Vaccine Hesitancy in LMICs: Approach and Applications

(updated August 1, 2023)

Contents

B1 Summary Statistics B1
B2 Main Analyses B5
B2.1 Regression Tables B6
B2.2 Transition Probability Matrices B6
B2.3 Stationary Distributions for Other Covariates B9
B3 Supplementary Analyses B13
B3.1 Alternative Sources and Measures B13
B3.2 Adjustments for Autocorrelated Errors B17
B3.2.1 Spatial Autocorrelation B17
B3.2.2 Temporal Autocorrelation B17
B3.3 Matched Analysis B21
B3.4 Cross-Level Interactions. B25
B4 SUNGEO R Package Code Examples B29

B1 Summary Statistics

Tables B1.1-B1.3 report summary statistics for all covariates used in our main analyses. These include both individual survey respondent-level information from the World Bank Group's High Frequency Phone Surveys (WBG HFPS), and contextual information from SUNGEO. The latter category draws on the following data sources:

- Constituency-Level Elections Archive (CLEA) (Kollman et al., 2022)
- DMSP-OLS Nighttime Lights Time Series (DMSP) (Hsu et al., 2015)
- Geo-referencing Ethnic Power Relations (EPR) (Wucherpfennig et al., 2011)
- ETOPO1 Global Relief Model (Bedrock) (ETOPO) (NOAA National Geophysical Data Center, 2009)
- Global Roads Open Access Data Set (gRoads) (CIESIN and ITOS, 2013)
- National Violence Monitoring System (NVMS) (Barron, Jaffrey and Varshney, 2016), preprocessed by xSub (Zhukov, Davenport and Kostyuk, 2019)
- Social Conflict Analysis Database (SCAD) (Salehyan et al., 2012), preprocessed by xSub (Zhukov, Davenport and Kostyuk, 2019)
- UCDP Georeferenced Event Dataset (UCDP-GED) (Sundberg and Melander, 2013), preprocessed by xSub (Zhukov, Davenport and Kostyuk, 2019)

Each observation in the dataset represents a household's survey response, observed across multiple survey rounds (unit of analysis is household-round). The SUNGEO covariates are observed at the second-tier administrative unit level (e.g. district) for Indonesia and Malawi, and at the first-tier administrative unit level for Kenya — where survey respondent geolocation is less precise (i.e. province-level).

We obtain estimates for these covariates by (a) using SUNGEO's R package to geocode the locations of survey sampling units, and (b) using this geolocation to spatially match each household to its parent administrative unit. Figure B1.1 illustrates this geoprocessing strategy, with the example of Indonesia.

Figure B1.1: Integration of Survey Data with Contextual Data on Violence, Elections and Road Infrastructure (Indonesia)

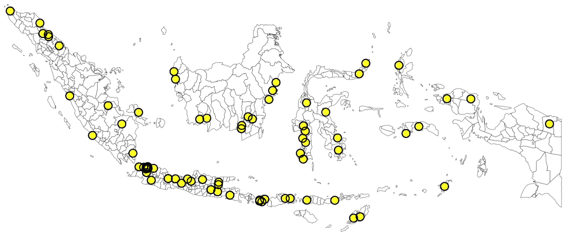

(a) Survey sampling units and level-2 administrative areas

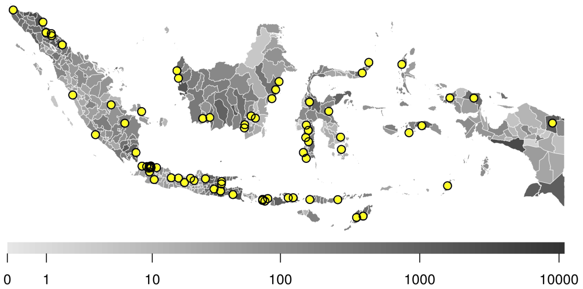

(b) Survey sampling units and violent events (percentile)

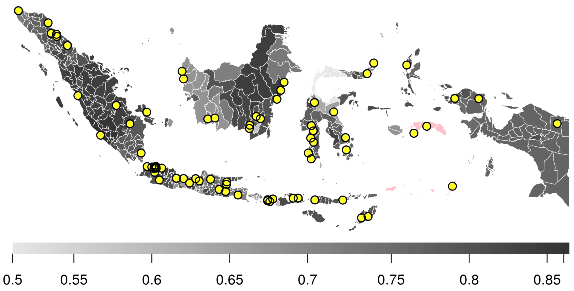

(c) Survey sampling units and electoral competitiveness (percentile)

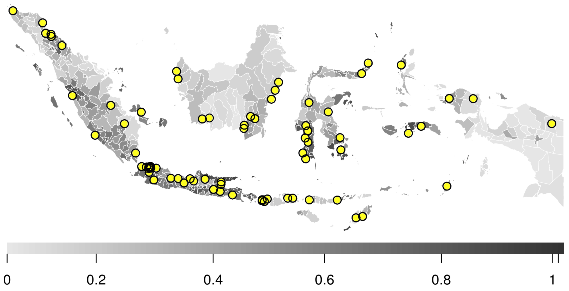

(d) Survey sampling units and local road density (percentile)

Variable	Range	Median	Mean	SD	Percentile (1, 99)	Source
Intent to take vaccine	(0, 1)	1.00	0.85	0.36	(0, 1)	WBG HFPS
Political violence (log)	(1.39, 9.24)	6.82	6.30	1.90	(1.39, 9.24)	NVMS
Electoral competitiveness	(0.81, 0.99)	0.97	0.94	0.05	(0.81, 0.99)	CLEA
Road density	(0, 0.57)	0.11	0.14	0.10	(0, 0.33)	gRoads
Night light intensity	(0.08, 63)	7.74	20.11	23.37	(0.11, 63)	DMSP
Slope of terrain	(0.02, 5.59)	1.23	1.68	1.65	(0.03, 5.02)	ETOPO
Ethno-linguistic fractionalization	(0, 0.62)	0.00	0.20	0.23	(0, 0.62)	EPR
Urbanization	(0, 1)	1.00	0.52	0.50	(0, 1)	WBG HFPS
Age	(12, 87)	44.00	44.35	9.64	(22, 70)	WBG HFPS
Sex	(0, 1)	0.00	0.37	0.48	(0, 1)	WBG HFPS

Table B1.1: Summary statistics for Indonesia data. 1847 households, 5 rounds.

Variable	Range	Median	Mean	SD	Percentile (1, 99)	Source
Intent to take vaccine	(0, 1)	1.00	0.87	0.34	(0, 1)	WBG HFPS
Political violence (log)	(-6.91, 8)	4.03	2.38	4.54	(-6.91, 8)	GED
Electoral competitiveness	(0.41, 0.88)	0.76	0.74	0.11	(0.41, 0.87)	CLEA
Road density	(0.03, 0.76)	0.33	0.31	0.21	(0.03, 0.76)	gRoads
Night light intensity	(0, 38.55)	0.32	5.70	12.59	(0.01, 38.55)	DMSP
Slope of terrain	(0.11, 4.81)	1.21	1.34	0.82	(0.11, 4.81)	ETOPO
Ethno-linguistic fractionalization	(0, 0.6)	0.01	0.13	0.19	(0, 0.6)	EPR
Urbanization	(0, 1)	0.00	0.47	0.50	(0, 1)	WBG HFPS
Age	(18, 98)	37.00	39.79	13.64	(19, 78)	WBG HFPS
Sex	(0, 1)	1.00	0.53	0.50	(0, 1)	WBG HFPS

Table B1.2: Summary statistics for Kenya data. 7616 households, 4 rounds.

Variable	Range	Median	Mean	SD	Percentile (1, 99)	Source
Intent to take vaccine	(0, 1)	1.00	0.74	0.44	(0, 1)	WBG HFPS
Political violence (log)	(-6.91, 5.56)	-6.91	-3.55	5.17	(-6.91, 5.56)	SCAD
Electoral competitiveness	(0.29, 0.98)	0.82	0.80	0.11	(0.47, 0.98)	CLEA
Road density	(0, 12.07)	0.13	0.47	1.87	(0, 12.07)	gRoads
Night light intensity	(0, 33.99)	0.33	7.34	11.15	(0, 33.99)	DMSP
Slope of terrain	(0, 5.15)	0.94	1.39	1.29	(0, 5.15)	ETOPO
Ethno-linguistic fractionalization	(0, 0.65)	0.00	0.04	0.13	(0, 0.65)	EPR
Urbanization	(0, 1)	0.00	0.33	0.47	(0, 1)	WBG HFPS
Age	(18, 86)	42.00	42.47	13.91	(21.96, 79)	WBG HFPS
Sex	(0, 1)	0.00	0.23	0.42	(0, 1)	WBG HFPS

Table B1.3: Summary statistics for Malawi data. 596 households, 2 rounds.

Table B1.4 reports survey sample attrition statistics. The "Attrition" column shows the number (and percent) of households who dropped out of the sample between each pair of rounds. We use an apostrophe (') to denote rounds that we excluded from our main analysis because their survey questionnaires did not include questions about vaccine hesitancy. To assess whether respondents who dropped out of the sample between rounds systematically differ from those who stayed, the table also includes standardized differences in means for the covariates in Tables B1.1-B1.3, including both the average standardized difference across all covariates, and the minimum and maximum values.

The statistics in this table suggest that sample attrition is potentially most problematic for our Kenya data, where drop rates are between 12 and 27 percent. On the other end of the spectrum are our data for Indonesia, where drop rates range from 3 to 9 percent. The largest standardized difference in means is half a standard deviation, for Malawi rounds 8 and 9 (the share of female respondents rose from 21 percent to 41 percent). Note, however, that we do not use data on Malawi's round 8 in our analyses. Most standardized differences, however, are well below .25 standard deviations, indicating that respondents who dropped out of these samples were quite similar on observables to those who remain.

Country	Rounds	Attrition	Std.Diff.(mean)	Std.Diff.(min)	Std.Diff.(max)
Indonesia	1',2'	221 (5%)	0.07	0.01	0.17
Indonesia	2',3'	138 (3%)	0.05	9e-05	0.09
Indonesia	3',4	204 (5%)	0.11	0.02	0.25
Indonesia	4,5	352 (9%)	0.07	0.03	0.15
Indonesia	5,6	310 (8%)	0.06	0.003	0.12
Indonesia	6,7	247 (7%)	0.07	5e-04	0.15
Kenya	1',2'	1098 (20%)	0.03	0.001	0.14
Kenya	2',3'	1694 (27%)	0.12	0.05	0.17
Kenya	3',4	1650 (26%)	0.08	0.003	0.18
Kenya	4,5	728 (12%)	0.15	0.01	0.22
Kenya	5,6	1160 (16%)	0.14	0.03	0.27
Kenya	6,7	855 (12%)	0.15	0.06	0.27
Malawi	5',8'	97 (6%)	0.18	0.04	0.35
Malawi	8',9	39 (3%)	0.26	0.14	0.49
Malawi	9,10	650 (42%)	0.09	0.02	0.17

Table B1.4: Survey sample attrition. Standardized difference (Std. Diff.) is $(\text{mean}(\mathbf{x}_s) - \text{mean}(\mathbf{x}_d))/\text{sd}(\mathbf{x}_s)$, where $\mathbf{x}_s$ are observed covariate values for respondents who stayed in the sample, and $\mathbf{x}_d$ are observed covariate values for respondents who dropped out of the sample. ' denotes that the survey round did not include questions about vaccine hesitancy.

B2 Main Analyses

The current section reports the full set of estimation results from the models used to generate the stationary distributions in Figures 2-4 in the main text. Out main model specification takes the following form:

$$\Pr(y_{i,t} = 1) = \text{logit}^{-1}\left[\mathbf{x_i}\theta_\mathbf{0} + y_{i,t-1} \cdot \mathbf{x_i}\gamma + \alpha_{j[i]} + \tau_t + \epsilon_{i,t}\right] \quad (1)$$

where y_{it} is equal to 1 if respondent i expressed an intent to obtain the Covid-19 vaccine in round t, and 0 if i did not express such an intent. $y_{i,t-1}$, is a first-order temporal lag of this indicator for the previous round.

$\mathbf{x_i}$ is a set of covariates, which include both respondent-level attributes (age, sex) and the social, economic and political characteristics of the respondent's local geographic environment (electoral competitiveness, exposure to political violence, road density, night light intensity, urbanization, ethnolinguistic fractionalization, geographic terrain). $\alpha_{j[i]}$ are fixed effects corresponding to the first-order administrative units (e.g. provinces) $j \in 1, \ldots, J$ in which households $i \in 1, \ldots, N$ are located.[1] τ_t are fixed effects for survey rounds $t \in 1, \ldots, T$. ϵ_{it} are robust standard errors, clustered by province and survey round.

θ_0 is the set of regression coefficients for households that had previously expressed no intent to get vaccinated ($y_{i,t-1} = 0$), and $\theta_1 = \theta_0 + \gamma$ is the set of coefficients for households that did express an intent to get vaccinated ($y_{i,t-1} = 1$). We use a logit link function to relate the covariates $\mathbf{x}$ to the corresponding transition probabilities $\Pr_{i,t}$("no" $\rightarrow$ "yes"), $\Pr_{i,t}$("yes" $\rightarrow$ "yes"). We use the predicted probabilities from this model to construct transition probability matrices under various counterfactual scenarios, and obtain stationary distributions of these transition matrices through eigenvalue decomposition.

To make the sample more closely resemble a simple random draw of each country's population, we weight each household observation with sampling weights provided by WBG HFPS. These weights are "based on the inclusion probabilities of the cell phones and landlines through which [respondents] can be reached," along with first-time and attrition non-response weighting adjustments, and calibration with auxiliary information on regional

[1] We omit this set of fixed effects for Kenya, because — due to the relative imprecision of spatial matching — there is no within-province variation in the geographic covariates.

population size, respondent sex, age group, and educational attainment (Flores Cruz, 2022).

B2.1 Regression Tables

Table B2.5 reports the raw $\hat{\theta}_0$ and $\hat{\gamma}$ estimates for equation (1), and model fit diagnostics.

B2.2 Transition Probability Matrices

Because the numerical estimates in Table B2.5 can be difficult to interpret on their own, Figures B2.6-B2.8 report transition probability matrices derived from the predicted probabilities of the models in Table B2.5. Figures B2.9-B2.11 show the stationary distributions of these transition matrices, based on an eigenvalue decomposition.

	no	yes
no	0.65	0.35
yes	0.04	0.96

Table B2.6: 2×2 right-stochastic matrix of vaccine intent (median household in Indonesia). Values represent predicted probabilities that a household originally in state i (row) transitioned to state j (column) across rounds. States include: 'no' (don't intend to take vaccine), 'yes' (do intend).

	no	yes
no	0.21	0.79
yes	0.11	0.89

Table B2.7: 2×2 right-stochastic matrix of vaccine intent (median household in Kenya). Values represent predicted probabilities that a household originally in state i (row) transitioned to state j (column) across rounds. States include: 'no' (don't intend to take vaccine), 'yes' (do intend).

Table B2.5: Regression coefficients. Outcome is expressed willingness to take vaccine. Fixed effect GLM (logit) coefficient estimates, clustered robust standard errors in parentheses.

	Indonesia	Kenya	Malawi
Response (t-1)	8.605	1.296	2.881
	(1.084)	(1.637)	(3.496)
Political violence	−0.183	−0.067	−0.269
	(0.321)	(0.052)	(0.138)
Electoral competitiveness	7.175	1.709	1.389
	(2.467)	(2.422)	(3.131)
Road density	−0.106	0.974	0.402
	(2.074)	(1.655)	(0.297)
Night light intensity	0.012	0.009	0.143
	(0.044)	(0.013)	(0.075)
Slope of terrain	0.090	0.061	−0.209
	(0.367)	(0.331)	(0.255)
Ethno-linguistic fractionalization	−1.844	1.230	−9.965
	(0.737)	(0.500)	(6.248)
Urbanization	−1.448	0.279	−0.798
	(0.583)	(0.271)	(1.341)
Age	0.011	0.001	0.000
	(0.025)	(0.017)	(0.026)
Sex	0.164	−0.272	−0.062
	(0.492)	(0.344)	(0.708)
Response (t-1)*Political violence	0.123	0.044	0.093
	(0.310)	(0.049)	(0.124)
Response (t-1)*Electoral competitiveness	−4.559	−1.113	−1.505
	(1.058)	(2.240)	(3.860)
Response (t-1)*Road density	4.326	−0.026	−0.534
	(0.560)	(1.521)	(0.296)
Response (t-1)*Night light intensity	−0.019	0.008	−0.040
	(0.028)	(0.015)	(0.084)
Response (t-1)*Slope of terrain	−0.026	0.100	0.089
	(0.254)	(0.386)	(0.265)
Response (t-1)*Ethno-linguistic fractionalization	2.375	−1.840	9.450
	(1.195)	(0.800)	(6.489)
Response (t-1)*Urbanization	1.396	−0.322	0.423
	(0.881)	(0.265)	(1.365)
Response (t-1)*Age	−0.066	−0.010	−0.006
	(0.032)	(0.007)	(0.027)
Response (t-1)*Sex	−0.936	0.461	−0.742
	(0.392)	(0.327)	(0.720)
Number of observations	3,383	14,906	566
RMSE	0.305	0.346	0.434
AIC	30,799,104	17,452,259	1,239,293
FE: Admin. units	X		X
FE: Survey rounds	X	X	X

	no	yes
no	0.40	0.60
yes	0.24	0.76

Table B2.8: 2×2 right-stochastic matrix of vaccine intent (median household in Malawi). Values represent predicted probabilities that a household originally in state i (row) transitioned to state j (column) across rounds. States include: 'no' (don't intend to take vaccine), 'yes' (do intend).

	no	yes
1	0.10	0.90

Table B2.9: Stationary distribution of 2×2 right-stochastic matrix (median household in Indonesia). Values represent long-term probabilities that a household ends up in each state, irrespective of initial distribution. States include: 'no' (don't intend to take vaccine), 'yes' (do intent to get vaccine).

	no	yes
1	0.12	0.88

Table B2.10: Stationary distribution of 2×2 right-stochastic matrix (median household in Kenya). Values represent long-term probabilities that a household ends up in each state, irrespective of initial distribution. States include: 'no' (don't intend to take vaccine), 'yes' (do intent to get vaccine).

	no	yes
1	0.28	0.72

Table B2.11: Stationary distribution of 2×2 right-stochastic matrix (median household in Malawi). Values represent long-term probabilities that a household ends up in each state, irrespective of initial distribution. States include: 'no' (don't intend to take vaccine), 'yes' (do intent to get vaccine).

B2.3 Stationary Distributions for Other Covariates

Figures B2.2-B2.4 report additional simulated stationary distributions, for all covariates besides those already discussed in the main text (competitiveness, violence and road density). Each sub-plot compares the stationary distribution under two counterfactual scenarios, where the focal covariate takes a particular value, while all other covariates are held constant at their median values.[2] For continuous covariates, the labels "high" and "low" correspond to the 99th and 1st percentiles (see Tables B1.1-B1.3). For example, Figure B2.2a suggests that the long-run probability of saying "yes" to the vaccine is higher for younger survey respondents: it is 0.96, on average, for respondents in the 99th age percentile of our Indonesian sample (70 years old), and 0.75 for those in the 1st percentile (18 or younger).

Figures B2.2-B2.4 highlight several patterns that are consistent across the three countries, as well as several points of cross-national variation. In all three countries, older respondents are more hesitant to take the vaccine. Respondents who lived in more ethnically fractionalized areas were also consistently more hesitant to get the vaccine than respondents in less fractionalized areas.

Other covariates vary in importance across countries. For example, while Kenyan and Malawian respondents expressed more willingness to obtain the vaccine in more economically active locations (as proxied by luminosity), this pattern did not hold in Indonesia. While urbanization appears to be a strong predictor of vaccine hesitancy in Indonesia and Malawi, we observe no such relationship in Kenya.

Finally, there are several covariates whose relationships to vaccine hesitancy vary across countries not only in statistical significance, but also in direction. While male respondents are more willing to take the vaccine in Indonesia and Malawi, the opposite is true in Kenya. While rugged terrain is correlated with greater vaccine acceptance in Indonesia and Kenya, the opposite is true in Malawi.

[2] We obtained 95% confidence intervals through a parametric bootstrap with 1000 iterations.

Figure B2.2: Additional Counterfactual Stationary Distributions, Indonesia

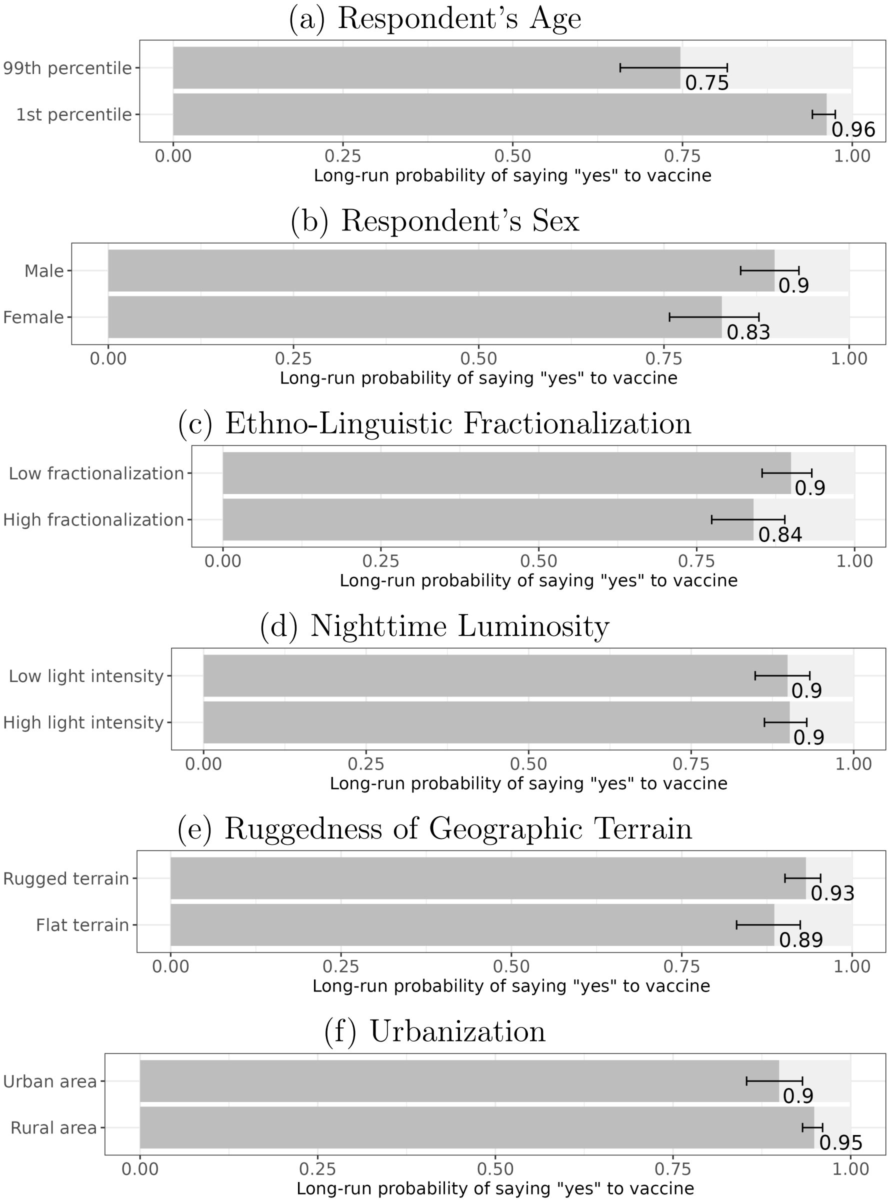

Figure B2.3: Additional Counterfactual Stationary Distributions, Kenya

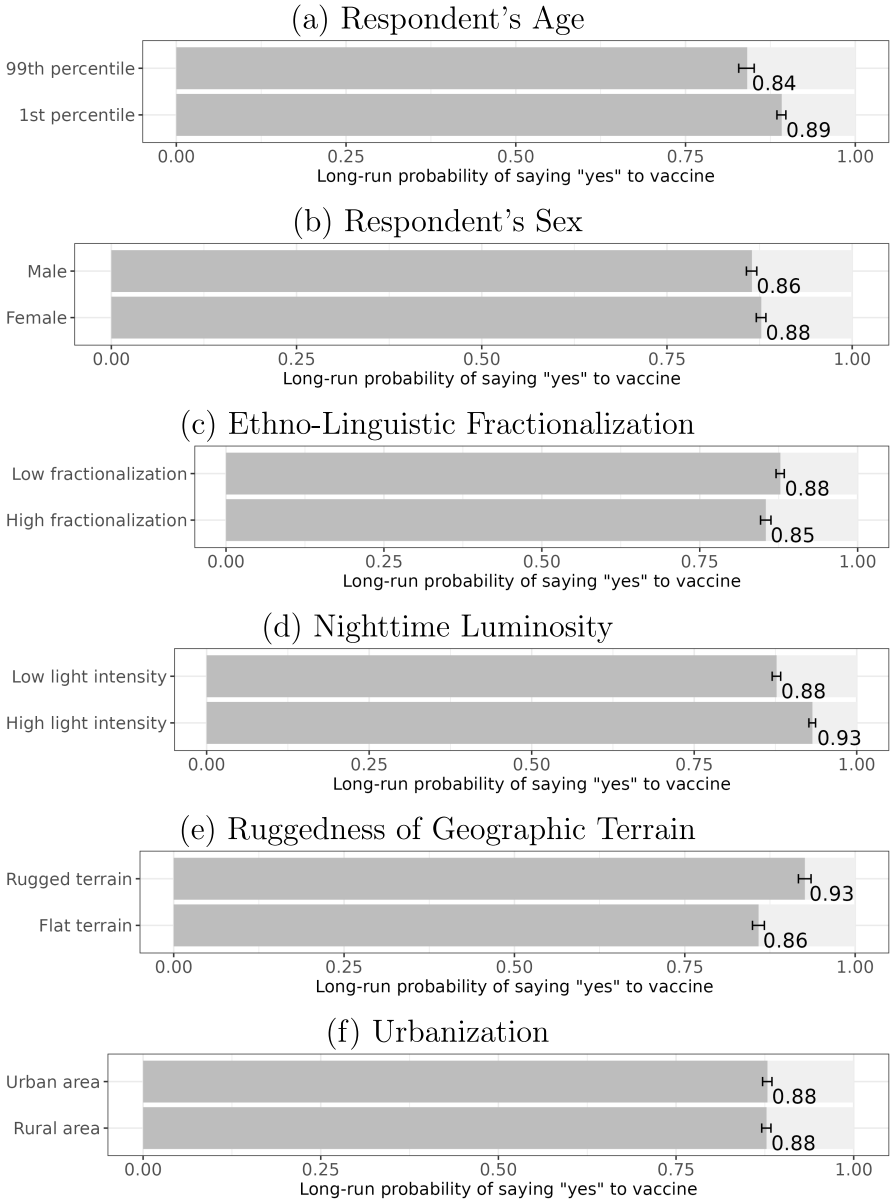

Figure B2.4: Additional Counterfactual Stationary Distributions, Malawi

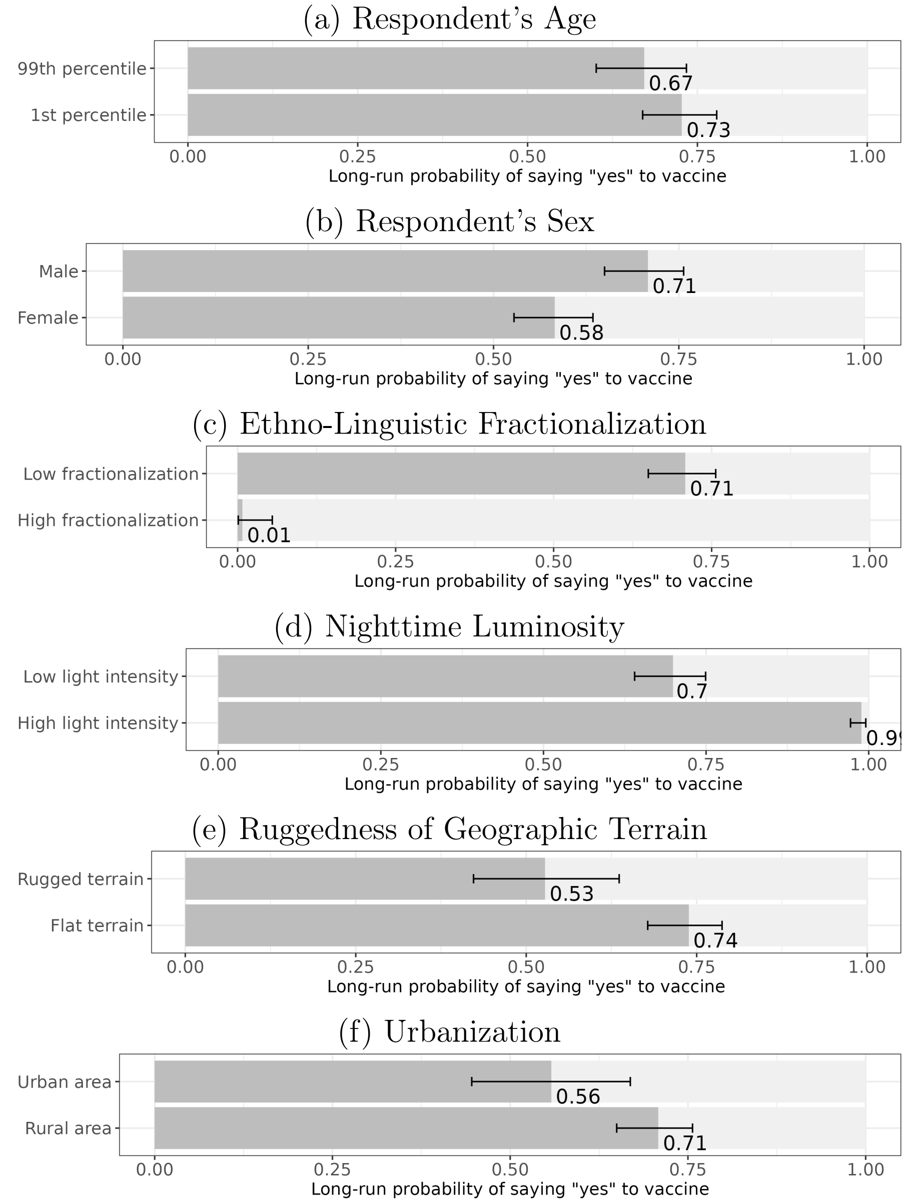

B3 Supplementary Analyses

B3.1 Alternative Sources and Measures

One of the advantages of SUNGEO as a platform for empirical research is the availability of multiple data sources and measures for the same theoretical constructs. For example, our main analysis employs specific measures of political violence and electoral competitiveness. Yet alternative data sources and measures for these variables do exist, each of which may represent a slightly different quantity of interest, and which may be subject to idiosyncratic forms of measurement error and bias. To gauge how sensitive our analyses are to these choices, we re-estimate the model in equation (1) with alternative data sources and measures, and report the resulting simulated stationary distributions below.

Figures B3.5-B3.7 report counterfactual stationary distributions with alternative data sources for political violence assembled by the Cross-National Data on Sub-National Violence (xSub) project, including the Armed Conflict Location and Event Data Project (ACLED), the National Violence Monitoring System (NVMS), the Social Conflict Analysis Database (SCAD), and the UCDP Georeferenced Event Dataset (UCDP-GED). Figures B3.8-B3.10 report stationary distributions for electoral competitiveness, using two alternative measures for lower-house parliamentary elections from the Constituency-Level Elections Archive: the Top Party Competitiveness Score (Top-1), and the Top-Two Party Competitiveness Score (Top-2). The Figures also report Akaike Information Criteria (AIC) for the models used for each simulation result, where lower values indicate lower deviance.

In the case of political violence, these analyses suggest that the direction of the relationship to vaccine hesitancy is mostly consistent across data sources (less violence → more willingness to take the vaccine), with several exceptions: UCDP-GED for Indonesia, SCAD for Kenya, and ACLED for Malawi. AIC statistics indicate that NVMS offers the best model fit in Indonesia, UCDP-GED offers the best fit for Kenya, and SCAD offers the best fit for Malawi. Consequently, these are the sources we employ in our main analyses.

In the case of electoral competitiveness, the two measures yield numerically similar estimates in all cases. AIC statistics indicate that — in 2 of 3 cases — Top-1 Competitiveness provides a superior model fit. For this reason, our main analyses employ the Top-1 measure.

Figure B3.5: Estimates with Alternative Political Violence Data Sources, Indonesia

(a) NVMS (AIC=30,846,246)

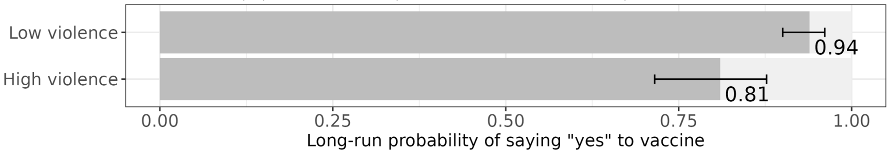

(b) ACLED (AIC=30,848,712)

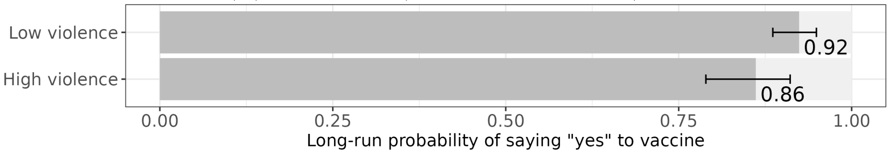

(c) UCDP-GED (AIC=30,876,146)

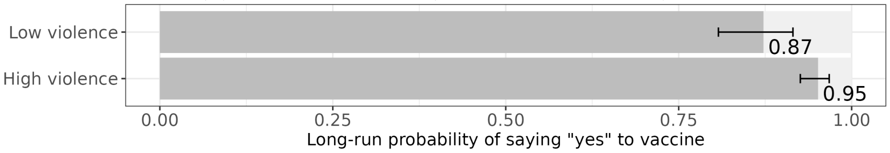

Figure B3.6: Estimates with Alternative Political Violence Data Sources, Kenya

(a) UCDP-GED (AIC=17,420,222)

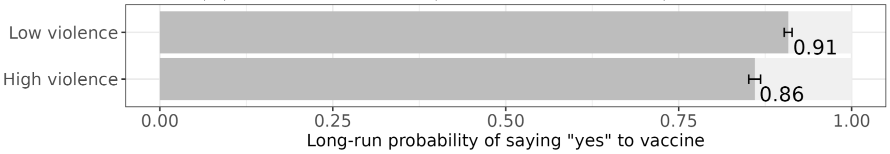

(b) ACLED (AIC=17,447,756)

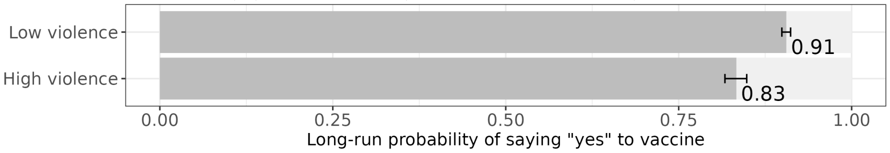

(c) SCAD (AIC=17,453,483)

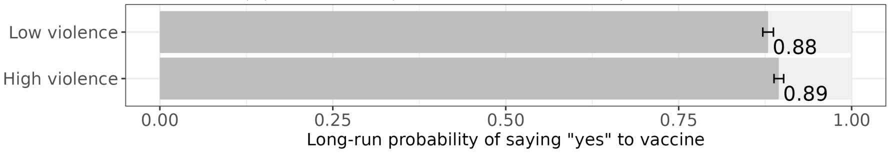

Figure B3.7: Estimates with Alternative Political Violence Data Sources, Malawi

(a) SCAD (AIC=1,238,644)

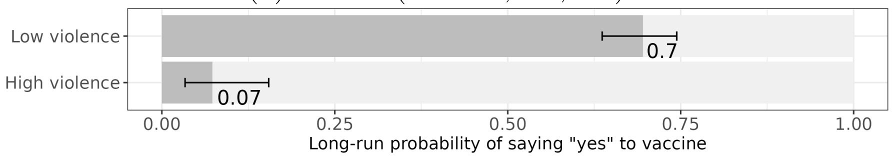

(b) ACLED (AIC=1,239,720)

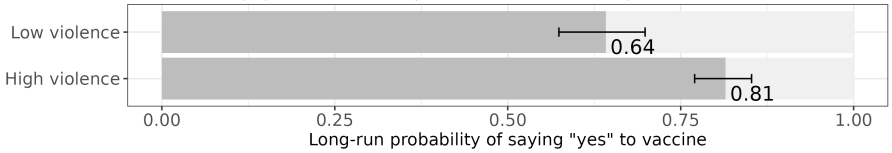

Figure B3.8: Estimates with Alternative Electoral Competitiveness Measures, Indonesia

(a) Top-2 Competitiveness (AIC=30,799,104)

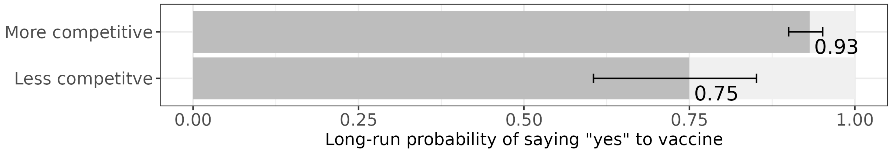

(b) Top-1 Competitiveness (AIC=30,846,246)

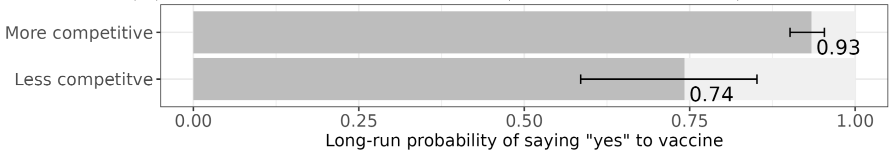

Figure B3.9: Estimates with Alternative Electoral Competitiveness Measures, Kenya

(a) Top-1 Competitiveness (AIC=17,420,222)

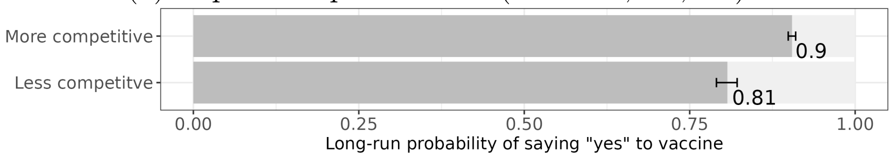

(b) Top-2 Competitiveness (AIC=17,452,259)

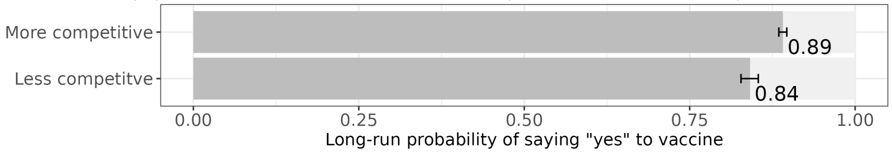

Figure B3.10: Estimates with Alternative Electoral Competitiveness Measures, Malawi

(a) Top-1 Competitiveness (AIC=1,238,644)

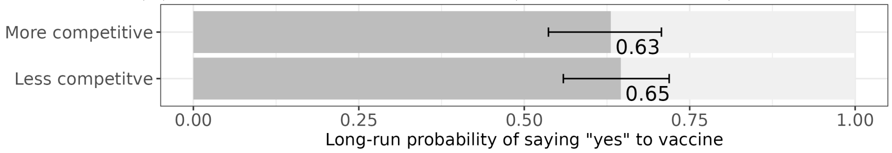

(b) Top-2 Competitiveness (AIC=1,239,293)

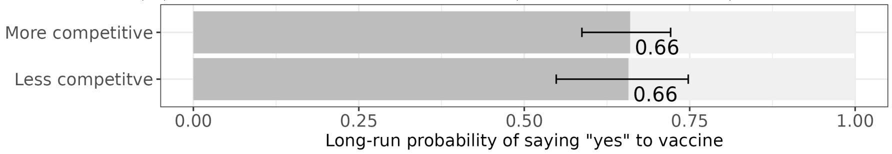

B3.2 Adjustments for Autocorrelated Errors

Our main analysis includes two-way clustered standard errors, which account for non-independence of survey observations in the same administrative unit and survey round. While conservative, this approach do not directly account for other types of potential spatial and temporal dependencies. First, within and across administrative areas, households in nearby locations may be more similar in their survey responses than households in more distant locations. Second, responses taken in consecutive survey rounds may be more similar to each other than responses from more distant, non-consecutive survey rounds. Both types of autocorrelation may lead to biased or inefficient estimates of relationships between local contextual factors and vaccine hesitancy. The current section reports estimates that directly adjust for spatially and termporally autocorrelated residuals.

B3.2.1 Spatial Autocorrelation

Figures B3.11-B3.13 report simulated stationary distributions with Conley (1999) standard error estimates, which correct for spatial correlation among respondent locations that fall within a set distance of each other. By way of a cutoff, we used the median distance from each primary sampling unit to its 5 nearest neighbors (80 km for Indonesia, 104 km for Kenya, 26 km for Malawi). This cutoff ensures that no sampling location is treated as a geographic isolate, and that each location is grouped with up to five others in its immediate geographic neighborhood. While estimates for Indonesia lose significance, the remaining results appear robust to this adjustment.

B3.2.2 Temporal Autocorrelation

To account for potential temporal dependence across rounds, Figures B3.14-B3.16 consider additional models with a nonparametric Driscoll-Kray time-series covariance matrix estimator, while is robust to general forms of cross-sectional and temporal dependence (in our case, up to one time lag). As with the Conley standard errors, this design yields wider confidence intervals than our main specification, particularly for Indonesia, indicating that autocorrelation may drive at least some of our results.

Figure B3.11: Estimates with Conley Standard Errors, Competitiveness

(a) Indonesia

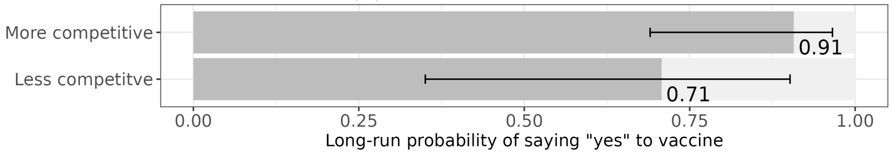

(b) Kenya

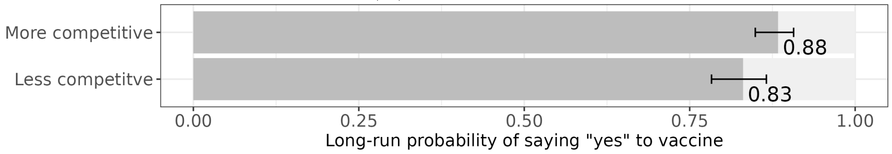

(b) Malawi

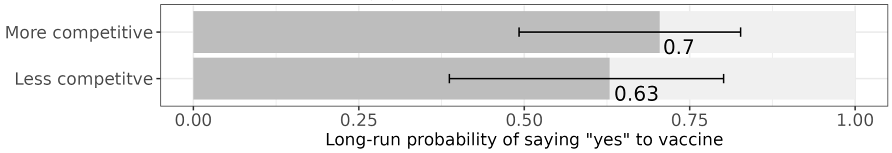

Figure B3.12: Estimates with Conley Standard Errors, Political Violence

(a) Indonesia

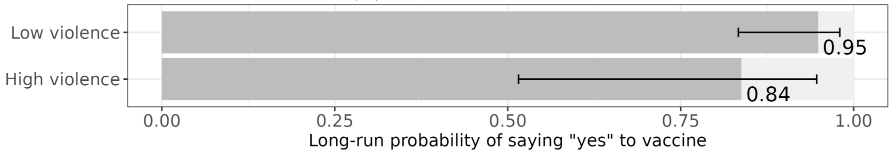

(b) Kenya

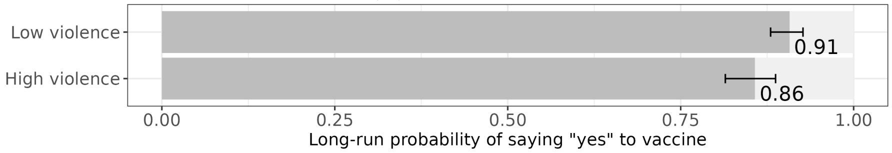

(b) Malawi

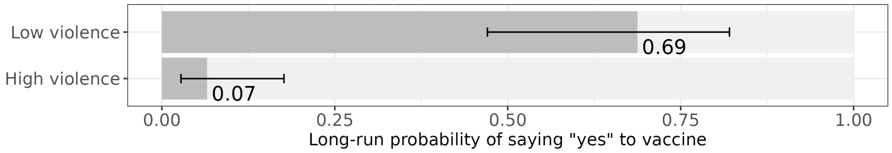

Figure B3.13: Estimates with Conley Standard Errors, Road Density

(a) Indonesia

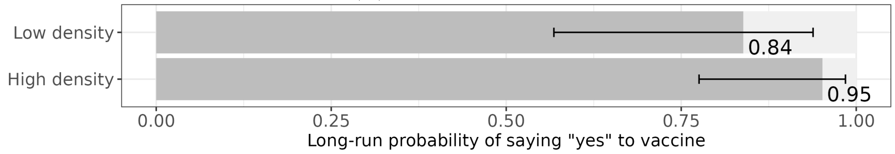

(b) Kenya

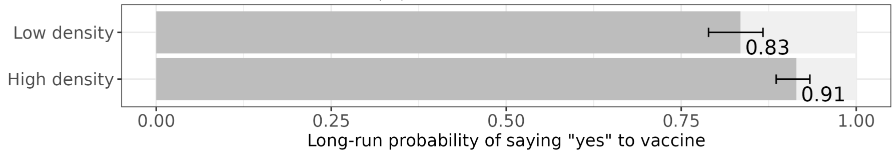

(b) Malawi

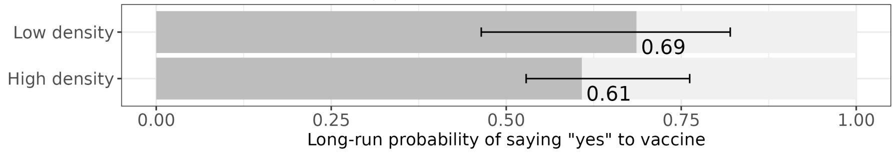

Figure B3.14: Estimates with Driscoll-Kray Standard Errors, Competitiveness

(a) Indonesia

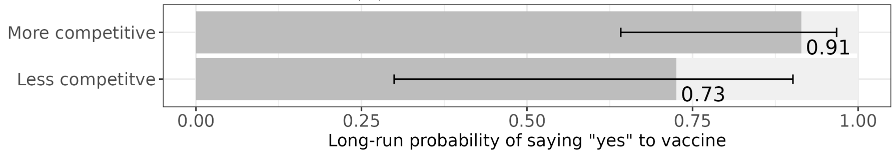

(b) Kenya

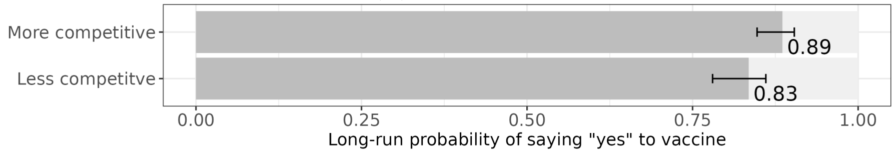

(b) Malawi

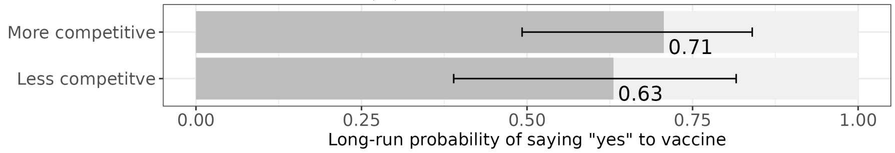

Figure B3.15: Estimates with Driscoll-Kray Standard Errors, Political Violence

(a) Indonesia

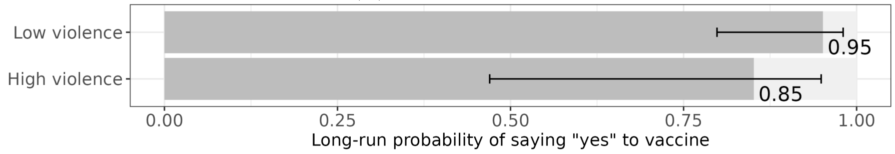

(b) Kenya

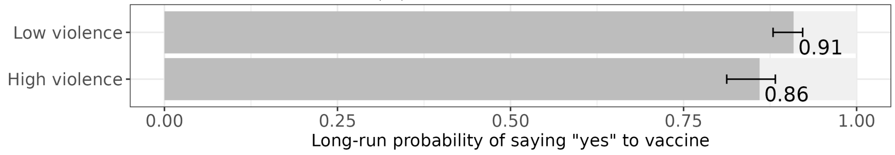

(b) Malawi

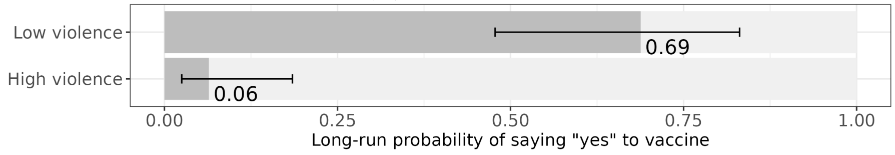

Figure B3.16: Estimates with Driscoll-Kray Standard Errors, Road Density

(a) Indonesia

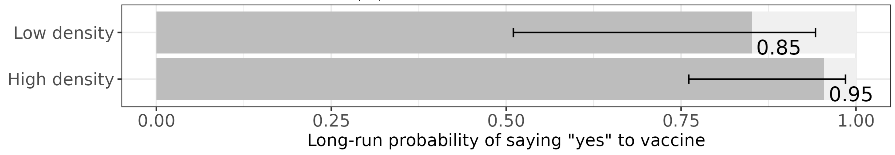

(b) Kenya

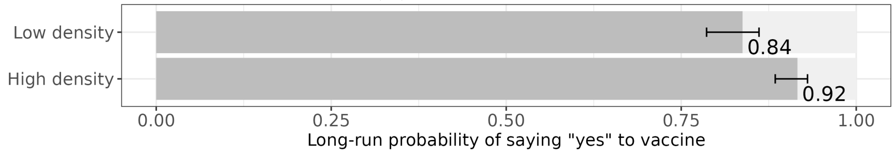

(b) Malawi

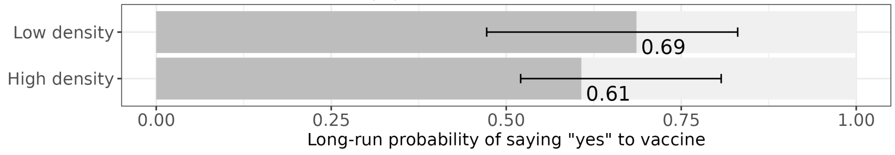

B3.3 Matched Analysis

One of several potential barriers to inference in our analysis is the possibility that our survey sample may be imbalanced on key contextual factors. Our main analyses use survey sampling weights (i.e. inverse probabilities of selection) to help approximate a random draw of the population. However, this re-weighting strategy does not address selection on dimensions beyond participant demographics. This problem may be particularly acute with respect to political violence. For example, survey sampling weights may account for the possibility that households in areas exposed to high levels of violence may be more difficult and costly for survey teams to reach, including by telephone. Yet even after accounting for their inclusion probability, these respondents may still differ from others in the sample in ways beyond their exposure to violence (e.g. differences in access to economic opportunities, access to infrastructure, differences in the local ethnolinguistic environment, type of geographic terrain). Some of these differences may also be relevant to vaccination, confounding our ability to asses the relationship between violence and vaccine hesitancy.

To alleviate some of these concerns, we used statistical matching to create re-weighted survey samples in which respondents with high exposure to violence were as similar as possible to respondents exposed to a lower level of violence. While matching is an adjustment-based solution that cannot facilitate causal inference without making quite onerous identifying assumptions — like the absence of unmeasured and unobserved confounders — this approach can reduce model dependence by down-weighting outliers and other influential observations, and preventing extrapolation outside the range of available data.

For each country, we applied three types of matching solutions: (1) propensity scores, which matches observations with a similar predicted probability of being selected into treatment (Rosenbaum and Rubin, 1983), (2) Mahalanobis distance, which seeks to minimize a scale-invariant distance between pre-treatment covariates, while taking into account correlation between the covariates (Sekhon, 2011), and (3) genetic matching, an extension of multivariate matching that uses an evolutionary search algorithm to determine the weight for each covariate (Sekhon and Diamond, 2013). We dichotomize the political violence variable for this purpose by labeling administrative units exposed to above-average levels of violence as "high violence" and those below the mean as "low violence." The matching

covariates are the same ones as in the matrix $\mathbf{x}$ in equation (1), excluding violence.

Table B3.12 summarizes covariate balance statistics before and after matching, for all three countries and all matching solutions. The metric we report here is standardized difference, or the absolute difference in means between "treated" (high violence) and "control" (low violence) units, divided by the standard deviation of the "treated" group. While there are no universally-accepted criteria for assessing improvements in balance, standardized bias of 0.25 and lower is a common standard in social science (Ho et al., 2007). The table reports averages of standardized bias, across all covariates.

For Indonesia, the matching solution with the greatest improvement in balance was Mahalanobis distance. For Kenya, propensity score matching out-performed the rest. In Malawi, no matching solution generated a major improvement in balance — standardized differences remained unacceptably high — with Mahalanobis distance in a narrow lead.

Figures B3.17-B3.19 report the simulated stationary distributions, re-estimated on each matched sample. While numerical estimates diverge significantly from those in the full sample, the direction of the relationship is — at least for Indonesia and Kenya — the same as before. The estimates for Malawi are a clear exception, although we urge caution in reading too deeply into them, given the significant remaining imbalance and small sample size reported in Table B3.12.

Table B3.12: SUMMARY OF COVARIATE BALANCE STATISTICS, PRE- AND POST-MATCHING.

Country	Method	Std.Dist.(pre)	Std.Dist.(post)	Improvement	Pairs
Indonesia	Genetic Matching	0.249	0.128	48.5%	635
Indonesia	Mahalanobis Distance	0.249	0.121	51.3%	635
Indonesia	Propensity Score	0.249	0.17	31.8%	635
Kenya	Genetic Matching	0.219	0.095	56.4%	9171
Kenya	Mahalanobis Distance	0.219	0.095	56.4%	9171
Kenya	Propensity Score	0.219	0.045	79.4%	9171
Malawi	Genetic Matching	0.596	0.463	22.3%	174
Malawi	Mahalanobis Distance	0.596	0.402	32.5%	174
Malawi	Propensity Score	0.596	0.564	5.3%	174

Standardized difference (Std. Diff.) is $\left|\frac{\text{mean}(T)-\text{mean}(C)}{\text{sd}(T)}\right|$.

Figure B3.17: Estimates with Matched Samples, Political Violence, Indonesia

(a) Genetic Matching

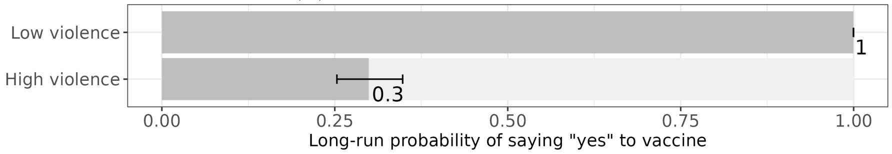

(b) Propensity Score Matching

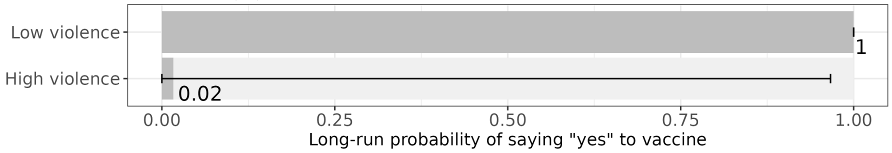

(c) Multivariate (Mahalanobis) Distance Matching

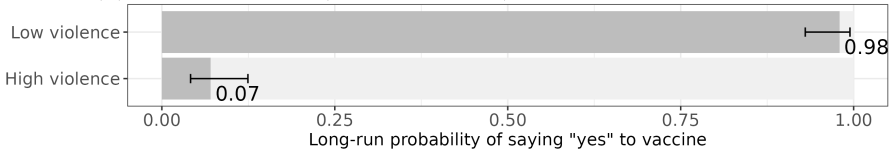

Figure B3.18: Estimates with Matched Samples, Political Violence, Kenya

(a) Genetic Matching

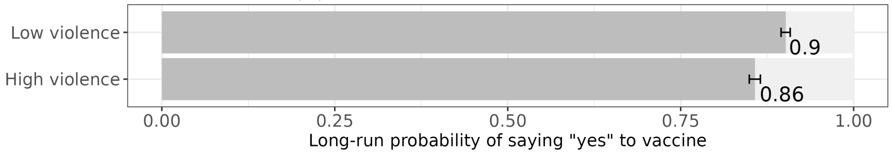

(b) Propensity Score Matching

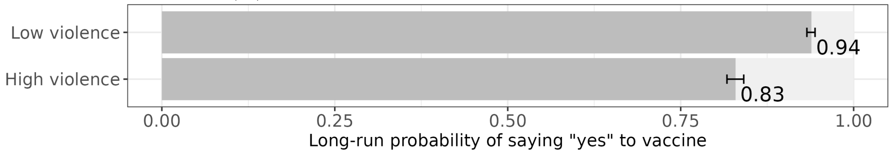

(c) Multivariate (Mahalanobis) Distance Matching

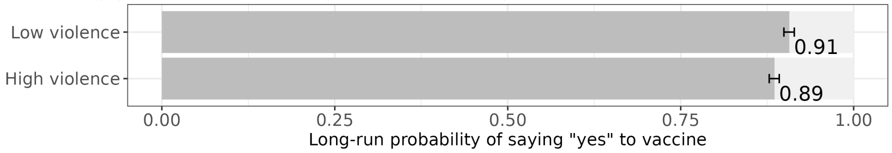

Figure B3.19: Estimates with Matched Samples, Political Violence, Malawi

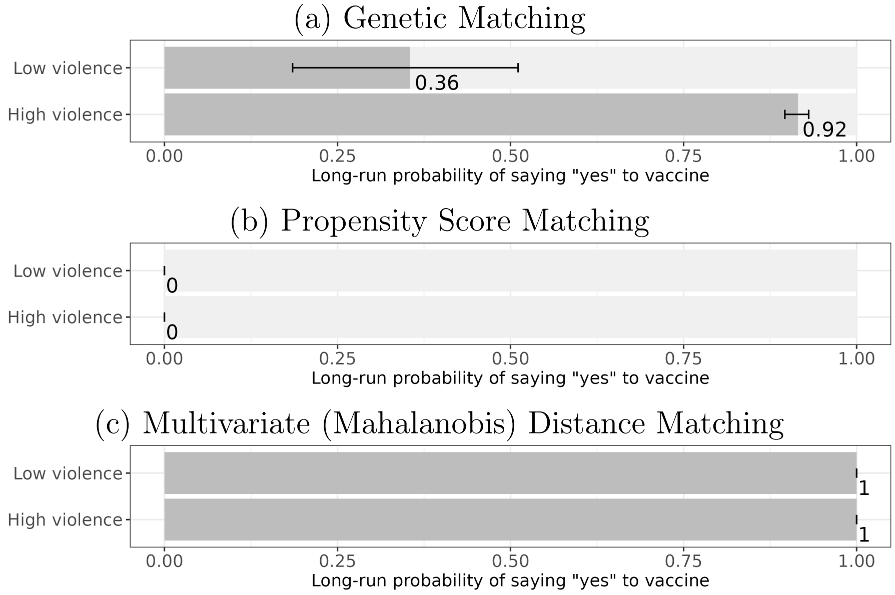

B3.4 Cross-Level Interactions

One possibility that our main model specification does not directly consider is that individual-level characteristics, such as age or sex, might interact with local contextual factors in ways that are relevant to vaccine hesitancy. For example, political violence can have profound demographic, social and economic effects on communities exposed to it. Armed conflict can shape the age distribution of exposed population, by lowering life expectancy and labor force participation rates among military-age males. It can also affect gender relations, in the direction of both empowerment and subjugation: war can increase women's participation in the labor force, albeit temporarily (e.g. World War II), yet it can also place new restrictions on women's social roles and economic opportunities (e.g. Syrian Civil War).

In our main analyses, we found that older respondents were more hesitant to take the vaccine, while male respondents were (generally) less vaccine-hesitant than female respondents (see Figures B2.2-B2.4). One possibility is that exposure to violence dampens these relationships, by reducing the salience of infectious disease as an immediate threat to life among directly affected groups (i.e. military-age males). An alternative possibility is that exposure to violence amplifies these relationships, creating starker differences in expressed opinion across age groups and sexes. To evaluate the relative merits of these two arguments, we adopt a slightly different model specification:

$$\begin{aligned}\Pr(y_{i,t} = 1) = \text{logit}^{-1}[&\text{Age}_{i,t}\beta_1 + \text{Sex}_{i,t}\beta_2 + \text{Violence}_i\beta_3 + (\text{Age}_{i,t} \cdot \text{Violence}_i)\beta_4 + \\ &(\text{Sex}_{i,t} \cdot \text{Violence}_i)\beta_5 + \mathbf{x_i}\theta + \alpha_{j[i]} + \tau_t + \epsilon_{i,t}]\end{aligned} \quad (2)$$

Figures B3.20 and B3.21 report several sets of estimates from these cross-level interaction models. The point values represent differences in predicted probabilities under two types of counterfactual scenarios (i.e. younger → older, female → male), broken down by level

of local exposure to violence. Formally, the quantities in Figure B3.20 are

$$\begin{aligned}\text{Upper row: } & \Pr(y_{i,t} = 1|\text{Age}_i = \text{99th percentile}, \text{Violence}_i = \text{Low}) \\ & - \Pr(y_{i,t} = 1|\text{Age}_i = \text{1st percentile}, \text{Violence}_i = \text{Low}) \\ \text{Lower row: } & \Pr(y_{i,t} = 1|\text{Age}_i = \text{99th percentile}, \text{Violence}_i = \text{High}) \\ & - \Pr(y_{i,t} = 1|\text{Age}_i = \text{1st percentile}, \text{Violence}_i = \text{High})\end{aligned}$$

and the quantities in Figure B3.21 are

$$\begin{aligned}\text{Upper row: } & \Pr(y_{i,t} = 1|\text{Sex}_i = \text{Male}, \text{Violence}_i = \text{Low}) \\ & - \Pr(y_{i,t} = 1|\text{Sex}_i = \text{Female}, \text{Violence}_i = \text{Low}) \\ \text{Lower row: } & \Pr(y_{i,t} = 1|\text{Sex}_i = \text{Male}, \text{Violence}_i = \text{High}) \\ & - \Pr(y_{i,t} = 1|\text{Sex}_i = \text{Female}, \text{Violence}_i = \text{High})\end{aligned}$$

With one exception (Kenya, age), the bulk of the evidence in Figures B3.20 and B3.21 suggests that exposure to violence dampens, rather than amplifies differences in vaccination hesitancy by age and sex. The associated differences in predicted probabilities of accepting the vaccine are numerically larger among respondents in low-violence areas than they are in high-violence areas. In high-violence areas, these differences are closer to zero.

Figure B3.20: Cross-Level Interaction: Age × Political Violence

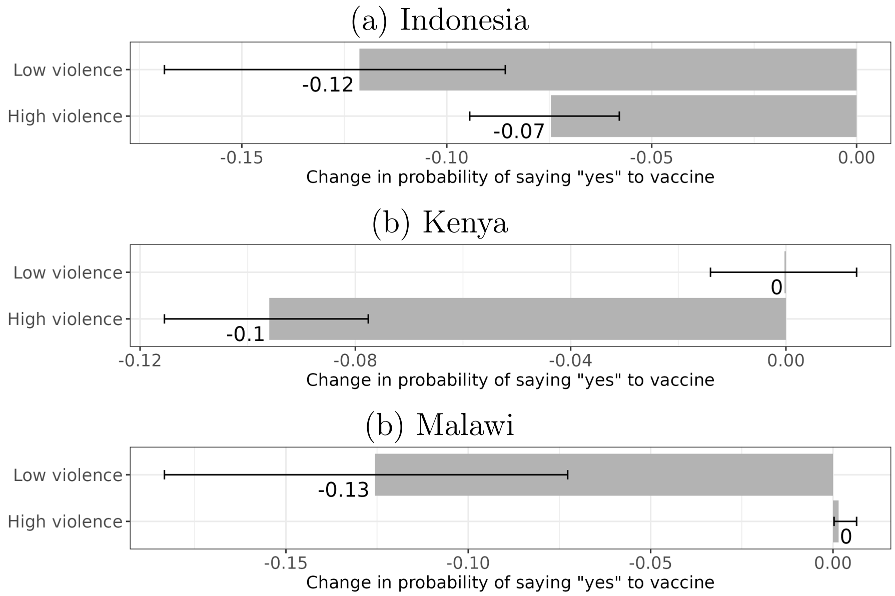

Counterfactual: respondent's age rises from 1st to 99th percentile.

Figure B3.21: Cross-Level Interaction: Sex × Political Violence

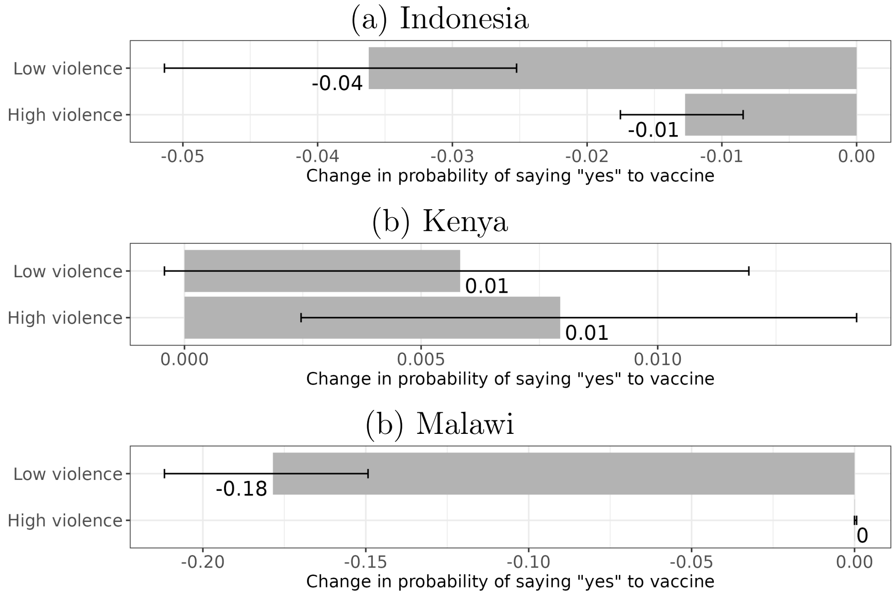

Counterfactual: respondent's sex switches from female to male.

B4 SUNGEO R Package Code Examples

The `SUNGEO` R package provides tools to download pre-processed data through our API, to implement several spatial transformation (change-of-support) methods, along with other data integration tasks. The package can be installed and loaded as follows:

```
# Install package
> install.packages("SUNGEO", dependencies = TRUE)

# Load package:
> library(SUNGEO)
```

Read help files for specific functions:

```
> ?get_data
> ?poly2poly_ap
> ?utm_select
```

Let's begin by browsing the data available through SUNGEO:

```
# Get list of all available data:
> info_1 <- get_info()
> info_1["summary"]
> info_1["topics"]
> info_1["geosets"]
```

Each of these commands will print to the console a list of available topics, and the countries and spatio-temporal units for which these data are available.

You may also narrow the search as follows:

```
# Get list of available data for a single country:
> info_2 <- get_info(country_names="Afghanistan")
> info_2["summary"]
> info_2["topics"]
> info_2["geosets"]

# Get list of available data for a single topic:
> info_3 <- get_info(topics="Elections:LowerHouse:CLEA")
> info_3["summary"]
> info_3["topics"]

# Get list of available data for a multiple countries and topics:
> info_4 <- get_info(
+            country_names=c("Afghanistan","Zambia"),
+            topics=c("Elections:LowerHouse:CLEA",
+                     "Events:PoliticalViolence:GED"))
> info_4["summary"]
```

Let's try downloading some data. For example, here is a query for a single country and single topic:

```
# Population data for Afghanistan:
> data_1 <- get_data(
+     country_name="Afghanistan",
+     topics="Demographics:Population:GHS")
[1] "Fetching..."
[1] "Combining..."
Time difference of 8.059215 secs
> str(data_1)
Classes 'data.table' and 'data.frame':  952 obs. of  34 variables:
...
```

Here is a query for a multiple countries and multiple topics:

```
# Population and ethnicity data for Albania and Moldova:
> data_2 <- get_data(
+     country_name=c("Albania","Moldova"),
+     topics=c("Demographics:Ethnicity:GREG",
+              "Demographics:Population:GHS"))
[1] "Fetching..."
[1] "Combining..."
Time difference of 1.481675 mins
> str(data_2)
Classes 'data.table' and 'data.frame':  1372 obs. of  39 variables:
...
```

You can modify the spatial units, time units and geographic boundary sets using the `space_unit`, `time_unit` and `geoset` options.

Let's now try to change the geographic support of a variable. We'll begin by loading some pre-installed spatial data for illustrative purposes: polygons representing German legislative districts and hexagonal grid cells:

```
# Load data
> data(clea_deu2009)
> data(hex_05_deu)

# Preview
> plot(clea_deu2009["geometry"])
> plot(hex_05_deu["geometry"],add=TRUE,border="grey")
```

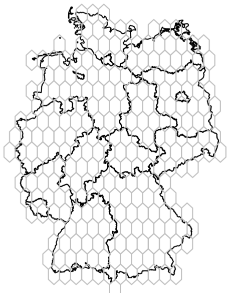

The grid cells appear generally smaller than legislative districts, and not nested. We can use the `nesting()` function to calculate scale and nesting metrics for the two sets of polygons. Let's calculate nesting metrics for a change of support from district to grid:

```
# Calculate all nesting metrics for a district-to-grid CoS
> nest_1 <- SUNGEO::nesting(
+                poly_from = clea_deu2009,
+                poly_to = hex_05_deu
+                )
> str(nest_1)
List of 12
 $ rs       : num 0.0252
 $ rn       : num 0.16
 $ rs_sym   : num -0.95
 $ rn_sym   : num -0.511
 $ rs_nn    : num 0.0252
 $ rn_nn    : num 0.16
 $ p_intact : num 0
 $ full_nest: num 0
 $ ro       : num -0.175
 $ gmi      : num 0.84
```

In this scenario, $RS = 0.025$, $RN = 0.16$, indicating disaggregation across non-nested units. Now let's check the opposite direction:

```
# Calculate all nesting metrics for a grid-to-district CoS
> nest_2 <- SUNGEO::nesting(
+                poly_from = hex_05_deu,
+                poly_to = clea_deu2009
+                )
> str(nest_2)
List of 12
 $ rs       : num 0.976
 $ rn       : num 0.67
 $ rs_sym   : num 0.953
 $ rn_sym   : num 0.511
 $ rs_nn    : num 0.97
 $ rn_nn    : num 0.528
 $ p_intact : num 0.588
 $ full_nest: num 0.302
 $ ro       : num 0.175
 $ gmi      : num 0.33
```

Here, $RS = 0.98, RN = 0.67$, indicating aggregation and more (but not perfect) nesting.

Let's try changing the geographic support for one variable (voter turnout) from constituencies to grid cells. While we strongly advise against transformations with such low RN and RS scores (see Zhukov et al., 2023), we implement this operation here for illustration.

```
# Areal interpolation
> out_1 <- poly2poly_ap(poly_from = clea_deu2009,
+                        poly_to = hex_05_deu,
+                        poly_to_id = "HEX_ID",
+                        varz = "to1"
+                        )
> str(out_1)
Classes 'sf' and 'data.frame':  257 obs. of  5 variables:
```

```
...

# Population-weighted interpolation
> data(gpw4_deu2010) # Load population raster
> out_2 <- poly2poly_ap(poly_from = clea_deu2009,
+                       poly_to = hex_05_deu,
+                       poly_to_id = "HEX_ID",
+                       varz = "to1",
+                       methodz = "pw",
+                       pop_raster = gpw4_deu2010)
> str(out_2)
Classes 'sf' and 'data.frame':  257 obs. of  5 variables:
...
```

The package also support more complex, model-based transformations. For example:

```
# Universal Kriging with one outcome variable and one covariate
> out_3 <- point2poly_krige(pointz = clea_deu2009_pt,
+                          polyz = clea_deu2009,
+                          yvarz = "to1",
+                          rasterz = gpw4_deu2010)
> str(out_3)
Classes 'sf' and 'data.frame':  16 obs. of  15 variables:
...
```

The `SUNGEO` package also has routines for batch geocoding of addresses (`geocode_osm()`, `geocode_osm_batch()`), overlays (`point2poly_simp()`), additional types of interpolation (`point2poly_tess()`, `line2poly()`), and other CoS methods. Please see the package help files for additional information.

References

Barron, Patrick, Sana Jaffrey and Ashutosh Varshney. 2016. "When Large Conflicts Subside: The Ebbs and Flows of Violence in Post-Suharto Indonesia.".

CIESIN and ITOS. 2013. "Global roads open access data set, version 1 (gROADSv1)." NASA Socioeconomic Data and Applications Center (SEDAC).
URL: *https://doi.org/10.7927/H4VD6WCT*

Flores Cruz, Ramiro. 2022. "High Frequency Phone Survey (HFPS) - Phase 2 Sampling Design, Weighting, and Estimation.".
URL: *https://microdata.worldbank.org/index.php/catalog/hfps*

Ho, Daniel E., Kosuke Imai, Gary King and Elizabeth A. Stuart. 2007. "Matching as nonparametric preprocessing for reducing model dependence in parametric causal inference." *Political Analysis* 15(3):199–236.

Hsu, Feng-Chi, Kimberly E Baugh, Tilottama Ghosh, Mikhail Zhizhin and Christopher D Elvidge. 2015. "DMSP-OLS radiance calibrated nighttime lights time series with intercalibration." *Remote Sensing* 7(2):1855–1876.

Kollman, Ken, Allen Hicken, Daniele Caramani, David Backer and David Lublin. 2022. *Constituency-Level Elections Archive*. Ann Arbor, MI: Center for Political Studies, University of Michigan.

NOAA National Geophysical Data Center. 2009. "ETOPO1 1 Arc-Minute Global Relief Model." NOAA National Centers for Environmental Information.

Rosenbaum, Paul R and Donald B Rubin. 1983. "The central role of the propensity score in observational studies for causal effects." *Biometrika* 70(1):41–55.

Salehyan, Idean, Cullen S Hendrix, Jesse Hamner, Christina Case, Christopher Linebarger, Emily Stull and Jennifer Williams. 2012. "Social conflict in Africa: A new database." *International Interactions* 38(4):503–511.

Sekhon, Jasjeet S. 2011. "Multivariate and Propensity Score Matching Software with Automated Balance Optimization: The Matching Package for R." *Journal of Statistical Software* 42(7):1–52.

Sekhon, Jasjeet S. and Alexis Diamond. 2013. "Genetic Matching for Estimating Causal Effects: A General Multivariate Matching Method for Achieving Balance in Observational Studies." *Review of Economics and Statistics* 95(3):932–945.

Sundberg, Ralph and Erik Melander. 2013. "Introducing the UCDP georeferenced event dataset." *Journal of Peace Research* 50(4):523–532.

Wucherpfennig, Julian, Nils B Weidmann, Luc Girardin, Lars-Erik Cederman and Andreas Wimmer. 2011. "Politically relevant ethnic groups across space and time: Introducing the geoepr dataset 1." *Conflict Management and Peace Science* 28(5):423–437.

Zhukov, Yuri M, Christian Davenport and Nadiya Kostyuk. 2019. "Introducing xSub: A New Portal for Cross-National Data on Sub-National Violence." *Journal of Peace Research* 56(4):604–614.

Zhukov, Yuri M, Jason S Byers, Marty A Davidson and Ken Kollman. 2023. "Integrating Data Across Misaligned Spatial Units." *Political Analysis* Forthcoming:1–17.
